# Divergence in salinity tolerance of northern Gulf of Mexico eastern oysters under field and laboratory exposure

**DOI:** 10.1093/conphys/coab065

**Published:** 2021-08-23

**Authors:** Danielle A Marshall, Sandra M Casas, William C Walton, F Scott Rikard, Terence A Palmer, Natasha Breaux, Megan K La Peyre, Jennifer Beseres Pollack, Morgan Kelly, Jerome F La Peyre

**Affiliations:** School of Renewable Natural Resources, Louisiana State University Agricultural Center, Baton Rouge, LA 70803, USA; School of Animal Sciences, Louisiana State University Agricultural Center, Baton Rouge, LA 70803, USA; School of Fisheries, Aquaculture, and Aquatic Sciences, Auburn University, Dauphin Island, AL 36528, USA; School of Fisheries, Aquaculture, and Aquatic Sciences, Auburn University, Dauphin Island, AL 36528, USA; Harte Research Institute for Gulf of Mexico Studies, Texas A&M University-Corpus Christi, TX, 78412, USA; Harte Research Institute for Gulf of Mexico Studies, Texas A&M University-Corpus Christi, TX, 78412, USA; US Geological Survey, Louisiana Fish and Wildlife Cooperative Research Unit, School of Renewable Natural Resources, Louisiana State University Agricultural Center, Baton Rouge, LA 70803, USA; Harte Research Institute for Gulf of Mexico Studies, Texas A&M University-Corpus Christi, TX, 78412, USA; Department of Biological Sciences, Louisiana State University, Baton Rouge, LA 70803, USA; School of Animal Sciences, Louisiana State University Agricultural Center, Baton Rouge, LA 70803, USA

**Keywords:** restoration, local adaptation, *Crassostrea virginica*, climate change, bivalves, Aquaculture, Editor: Dr Steven Cooke

## Abstract

The eastern oyster, *Crassostrea virginica*, is a foundation species within US Gulf of Mexico (GoM) estuaries that has experienced substantial population declines. As changes from management and climate are expected to continue to impact estuarine salinity, understanding how local oyster populations might respond and identifying populations with adaptations to more extreme changes in salinity could inform resource management, including restoration and aquaculture programs. Wild oysters were collected from four estuarine sites from Texas [Packery Channel (PC): 35.5, annual mean salinity, Aransas Bay (AB): 23.0] and Louisiana [Calcasieu Lake (CL): 16.2, Vermilion Bay (VB): 7.4] and spawned. The progeny were compared in field and laboratory studies under different salinity regimes. For the field study, F1 oysters were deployed at low (6.4) and intermediate (16.5) salinity sites in Alabama. Growth and mortality were measured monthly. Condition index and *Perkinsus marinus* infection intensity were measured quarterly. For the laboratory studies, mortality was recorded in F1 oysters that were exposed to salinities of 2.0, 4.0, 20.0/22.0, 38.0 and 44.0 with and without acclimation. The results of the field study and laboratory study with acclimation indicated that PC oysters are adapted to high-salinity conditions and do not tolerate very low salinities. The AB stock had the highest plasticity as it performed as well as the PC stock at high salinities and as well as Louisiana stocks at the lowest salinity. Louisiana stocks did not perform as well as the Texas stocks at high salinities. Results from the laboratory studies without salinity acclimation showed that all F1 stocks experiencing rapid mortality at low salinities when 3-month oysters collected at a salinity of 24 were used and at both low and high salinities when 7-month oysters collected at a salinity of 14.5 were used.

## Teaser

Using field and laboratory studies, we provide evidence of adaptation to local salinity regimes in the progenies of US Gulf of Mexico eastern oysters from estuaries differing substantially in annual mean salinity. Determining specific adaptations of oysters to local environmental conditions is important for conservation, restoration and the expanding aquaculture industry.

## Introduction

The eastern oyster, *Crassostrea virginica* (hereafter, oyster), is a foundation species that provides critical ecosystem services and supports an economically valuable fishery and aquaculture industry within US Gulf of Mexico (GoM) estuaries (Coen *et al.,* 2007; Nevins *et al.,* 2014; Volety *et al.,* 2014; La Peyre *et al.,* 2019a; NOAA, 2020). However, declines in oyster populations have been driven by factors such as over-harvest and changing estuarine environmental quality (Wilber, 1992; Beck *et al.,* 2011; Beseres Pollack *et al.,* 2011; Petes *et al.,* 2012; Soniat *et al.,* 2013). Altered salinity regimes from riverine and coastal management (e.g. river flow management, marsh management) and climate change (e.g. rising temperature, sea level rise) can significantly impact oysters, either directly by exceeding their physiological tolerances (La Peyre *et al.,* 2013; Rybovich *et al.,* 2016; Casas *et al.,* 2018; Lavaud *et al.,* 2021) or indirectly through impacts on their food, disease or predation (Soniat *et al.,* 2004; Riekenberg *et al.,* 2015; La Peyre *et al.,* 2019b) within GoM estuaries. As changes from management and climate are expected to continue to impact estuaries, understanding how local oyster populations might respond and identifying populations with specific adaptations to more extreme changes could inform resource management, including restoration and aquaculture programs.

The survival, growth and reproduction of oysters are driven predominantly by salinity and temperature (Shumway, 1996; Bayne, 2017). Salinity, in particular, has proven critical in influencing all aspects of oyster biology, despite oysters’ wide tolerance to varying and wide-ranging salinity regimes. Across the GoM, oysters survive in areas with mean annual salinities ranging from ~5 to over 35, providing a unique opportunity to examine population-specific adaptations and response to changing salinities (Breuer, 1962; Lowe *et al.,* 2017). Within Louisiana, several field studies have suggested the presence of genetically differentiated populations with respect to low salinity (<5) tolerance as well as dermo disease resistance at higher salinity (>15) (Leonhardt *et al.,* 2017; La Peyre *et al.,* 2019b). For example, juveniles reared in the hatchery from the spawn of wild-collected adults differed in mortality when out-planted, depending on the parents’ site of origin (Leonhardt *et al.,* 2017), mirroring evidence for adaptation to ‘local’ environmental conditions among oyster populations in other regions (e.g. Dittman *et al.,* 1998; Burford *et al.,* 2014).

Estuaries across the GoM differ in morphology and inflows, resulting in estuaries with markedly different environmental conditions including current and predicted salinity regimes (Orlando Jr *et al.,* 1993; Montagna *et al.,* 2013). Moreover, changes in salinity across GoM estuaries associated with climate change are expected to vary by region. Despite uncertainty associated with regional climate models, according to the US Global Change Research Program, the southwestern part of the USA, including Texas, will become hotter and drier in the 21st century, compounding pre-existing water deficits (Kloesel *et al.,* 2018). In contrast, across the southeastern part of USA, the frequency and intensity of precipitation events have been increasing (Powell and Keim, 2015; Carter *et al.,* 2018; Kloesel *et al.,* 2018), with effects of decreasing salinity particularly evident in Louisiana estuaries with increasing riverine flow, exacerbated by freshwater diversions (Soniat *et al.,* 2013; Wang *et al.,* 2017; Lavaud *et al.,* 2021). Superimposed on those predicted changes are increasing frequency and intensity of extreme climatic events (e.g. hurricanes, floods, droughts, heat waves) affecting water quality and further increasing salinity variability and extremes (Biasutti *et al.,* 2012; Wetz and Yoskowitz, 2013; Prein *et al.,* 2017). To date, however, limited effort has been exerted to assess the tolerance of oysters to extreme salinities or to evaluate local adaptation of oysters to the diverse environmental conditions of GoM estuaries.

Local adaptation occurs whenever resident genotypes outperform nonresident genotypes under local conditions (Kawecki and Ebert, 2004). Local adaptation was historically assumed to be rare in marine invertebrates because many, like *C. virginica*, have planktonic larvae and high gene flow that should tend to erase population differences. However, recent work has demonstrated that adaptive differences can occur even in species with high levels of gene flow (Sanford and Kelly, 2011). Local adaptation to salinity would imply that populations of oysters in GoM estuaries will respond uniquely to changing environments and that the outcome of restoration efforts or aquaculture production at a particular location would depend on the source population used in supportive breeding efforts. Further, understanding the range of oyster population responses to changing environmental conditions provides insight into their ability to respond and adapt to changing climate and will inform efforts to manage oysters for long-term sustainability. We therefore tested whether salinity, as an agent of divergent selection, was driving local adaptation in oysters. Using a combination of laboratory and field experiments, we examined components of fitness, across a range of salinities, of the progeny of oysters from four GoM oyster populations originating from estuaries with differing salinity regimes.

## Methods

### Oyster stocks

**Figure 1:**
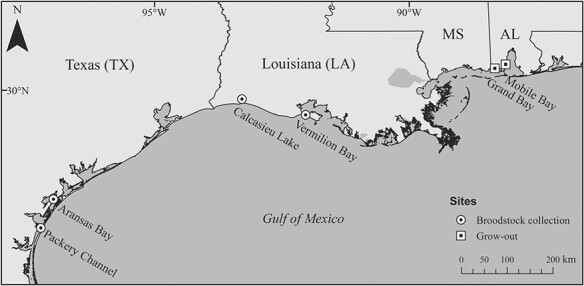
Map of wild broodstock collection sites [circle with dot: PC and AB in Texas (TX) and CL and VB in Louisiana (LA)] and progeny-testing grow-out sites [square with dot: Grand Bay and Mobile Bay, Alabama (AL)].

Between December 2017 and January 2018, 200 wild adult oysters were collected from each of the two Louisiana public oyster grounds, Calcasieu Lake (CL; 29° 50′ 58′′ N, 93° 17′ 1′′ W) and Vermilion Bay (VB; 29° 34′ 47′′ N, 92° 2′ 4′′ W). The oysters were placed in bags on an adjustable long line system (ALS, BST Oyster Co., Cowell, South Australia) at the Louisiana Sea Grant Oyster Research and Demonstration Farm in Grand Isle, Louisiana (29° 14′ 20′′ N, 90° 0′ 11′′ W) to ensure gonad development for spawning (i.e. salinity, >10) (Fig. 1). These two estuaries are separated by more than 100 km and have different salinity regimes with annual means [± standard deviation (SD), *N* = 10] of 16.2 ± 2.8 for CL and 7.4 ± 1.6 for VB from 2009 to 2018. Hydrological data associated with monthly oyster dredging by Louisiana Department of Wildlife and Fisheries at the respective public oyster grounds were used to calculate annual salinity and temperature (Supplemental Figs 1, 2). The Louisiana-collected broodstocks were conditioned for 8–9 months at Grand Isle before being transferred to the Auburn University Shellfish Laboratory (AUSL) hatchery in Dauphin Island, Alabama, for spawning. The 2009–2018, January to August, mean monthly salinity at the Grand Isle broodstock conditioning site was 18.0 ± 6.2 (USGS gage 073802516) and very close to the CL mean salinity, but about 10 above VB mean salinity (Supplemental Fig. 1).

In August 2018, 150 oysters were collected from two estuarine sites in southeastern Texas, Packery Channel (PC; 27° 37′ 38′′ N, 97° 13′ 59′′ W) and Aransas Bay (AB; 28° 7′ 38′′ N, 96° 59′ 8′′ W) (Fig. 1). The PC site has an annual mean salinity (2009–2018, *N* = 10) of 35.5 ± 5.1 and is within the Upper Laguna Madre adjacent to PC, a channel that links Laguna Madre (a hypersaline lagoon) with the GoM. AB has a mean salinity of 23.0 ± 6.9. Hydrological data associated with Texas Parks and Wildlife Department fisheries-independent monitoring program were used to calculate annual salinity and temperature within ~ 4 km of collection site (Martinez-Andrade, 2018, Supplemental Figs 1, 2). The Texas-collected broodstocks were directly transferred to AUSL hatchery for spawning.

In August 2018, each of the four broodstock groups was naturally induced to spawn at the AUSL hatchery by increasing water temperature, with individual oysters kept in separate 3-l containers to allow control of fertilization (Wallace *et al.,* 2008). Gametes were collected from spawning individuals, and the eggs from each female were fertilized by sperm from each male and pooled (Table 1). As a standard hatchery practice, micro-cultch material was used to set pediveliger larvae to produce single oyster spat in a recirculating downweller system. After 72 h, oyster spat were placed and maintained in upwelling nursery systems, until they reached ~6 mm in shell height, at which time they were placed in bags and moved to an AUSL-permitted grow-out site at Bayou Sullivan, Alabama (30° 21′ 52′′ N, 88° 12′ 57′′ W) for further growth. Mean daily salinity at the AUSL hatchery from the time of spawning to the time oyster spat reached 6 mm and were field deployed was 20.5 ± 5.7 and within the optimal range for larval rearing, setting and spat grow-out.

**Table 1:** Spawning date, stock designation, number of naturally spawned females and males and number of fertilized eggs used to produce the progenies of Texas oysters from PC and AB broodstocks and of Louisiana oysters from CL and VB broodstocks

Spawn date	Stock	Females	Males	Fertilized eggs
22 August 2018	AB	5	3	12 920 000
22 August 2018	VB	11	7	104 620 000
24 August 2018	AB	9	8	22 400 000
28 August 2018	CL	38	12	85 820 000
29 August 2018	PC	21	15	109 000 000

### Salinity tolerance during field exposure

#### Experimental design and methods

The performance of oysters from each of the four stocks was compared in the field at a low-salinity site (Mobile Bay, Bama Bay Oyster Company, AL, 30° 26′ 29′′ N, 88° 06′ 15′′ W) and intermediate-salinity site (Grand Bay, Grand Bay Oyster Park, AL, 30° 22′ 15′′ N, 88° 19′ 0′′ W) over 10 months (Fig. 1; December 2018–October 2019), with no high-salinity site available in Alabama. In December 2018, 400 oysters from each stock (initial shell heights given in Supplemental Table 1) collected from Bayou Sullivan grow-out site (slightly lower salinity than Grand Bay Oyster Park) were placed into four ALS bags (100 oysters per bag) and suspended underwater on an ALS at the Mobile Bay and Grand Bay sites (4 bags × 4 stocks × 2 sites). The bags were fully closed, and any potential predators (e.g. juvenile crabs) were removed during monthly sampling; mortality was mainly due to stressful abiotic conditions (e.g. salinity, temperature) or infection by *Perkinsus marinus*, the protistan parasite causing dermo disease in diploid eastern oysters based on past findings (Casas *et al.,* 2017; Leonhardt *et al.,* 2017; La Peyre *et al.,* 2019b). Every month, the shell heights of 25 oysters sampled haphazardly from each bag were measured using a digital caliper (Mitutoyo USA, Aurora, Illinois). Interval growth rates (mm month^−1^) were calculated by subtracting mean shell height from each bag at selected sampling time from the mean shell height of the same bag from the previous selected sampling time and standardizing to a 30-day month. Growth rates of each stock were obtained for time intervals ending in the April, July and October sampling dates when oysters were collected to measure condition index (CI) and *P. marinus* infection intensity as described below (Supplemental Table 2). These months were selected to follow usual changes of CI and *P. marinus* infection intensity previously reported in GoM oysters (Wadsworth *et al.,* 2019; La Peyre *et al.,* 2019b). Moreover, the biologically relevant intervals selected reflected periods of decreasing and low salinities (December–April), increasing salinities (April–July) and highest salinities (July–October) at two Alabama sites where the performance of several stocks of Alabama and Louisiana oysters were previously compared (Casas *et al.,* 2017) while keeping the number of intervals to a minimum for clarity and statistical analysis expediency. The numbers of live and dead oysters in each bag were counted and dead oysters were removed. Interval and cumulative mortality were then calculated following the method of Ragone Calvo *et al.* (2003).

In April, July and October, 20 oysters of each stock from each site (5 oyster bag^−1^) were collected and transported to the Louisiana State University Agricultural Center Animal and Food Sciences laboratory building (LSU AFL), in Baton Rouge, to measure *P. marinus* infection intensity and the CI by dry weight (CI_DW_). *Perkinsus marinus* infection intensity (i.e. the number of parasites per gram of oyster wet tissue) was determined using the whole-oyster procedure described by Fisher and Oliver (1996) and modified by La Peyre *et al.* (2003) except that oyster tissues were suspended in 0.2 μm filtered seawater at a concentration of 0.25 g ml^−1^ instead of alternative Ray’s fluid thioglycollate medium (ARFTM) during the homogenization step and 1 ml of the homogenate was then added to 9 ml of ARFTM (La Peyre *et al.,* 2019b). For each oyster, a 10-ml tissue homogenate aliquot was dried at 65°C for 48 h and the whole oyster dry meat weight (DW) was calculated based on the volume of homogenized tissue. The CI_DW_ was calculated as the ratio of DW to the whole oyster wet weight minus its shell wet weight (i.e. filled shell cavity weight) multiplied by 100 (Abbe and Albright, 2003) and indicates how well an oyster uses the shell cavity available for somatic and gonadal tissue growth and reflects physiological or nutritive status (Rainer and Mann, 1992).

Salinity and temperature were measured on oyster sampling days at the Mobile Bay and Grand Bay field sites using YSI Pro30 conductivity meter (YSI Inc., Yellow Springs, Ohio). Daily temperature and salinity data for Mobile Bay were obtained from a location (30° 25′ 33′′ N, 88° 6′ 10′′ W) less than 2 km south of the Mobile Bay field site. Mean daily salinity and temperature measurements at the Mobile Bay field site were 0.9 ± 1.3 above, and <0.1°C above mean measurements from the more southern site on the days oysters were sampled. Measurements at the Mobile Bay field and the more southern site on sampling days were highly correlated (Pearson’s Correlation; salinity: rho = 0.983, *P* < 0.0001; temperature: rho = 0.971, *P* < 0.0001) over the study period. Daily salinity and temperature data for Grand Bay were obtained from Point Aux Chenes station (PAC; 30° 20′ 54′′ N, 88° 25′ 6′′ W, Grand Bay National Estuarine Research Reserve, http://cdmo.baruch.sc.edu/dges) 10 km west of the Grand Bay field site. Mean daily salinity and temperature measurements at the Grand Bay field site were 0.6 ± 2.0 above, and 1.2 ± 0.3°C below mean PAC measurements on the days oysters were sampled. Measurements at PAC and Grand Bay field site on sampling days were highly correlated (Pearson’s Correlation; salinity: rho = 0.820, *P* = 0.013; temperature: rho = 0.981, *P* < 0.0001) over the study period. Mean salinity and temperature at the Mobile Bay and Grand Bay sites for the December –April, April–July and July–October intervals were calculated using only data when present at both sites on the same days.

#### Statistical analyses

Differences in salinity and temperature among sites and sampling intervals (December–April, April–July, July–October) were determined using a two-factor analysis of variance (ANOVA) test. Differences in mortalities among stocks and between sites at sampling intervals were compared using two-factor ANOVA tests; the random effect bag did not improve the model fit. Shell heights among stocks and between sites at the time of deployment in December 2018 were compared using a two-factor ANOVA test. Because significant differences in shell height were found between stocks at the time of deployment, growth rates calculated for the December–April, April–July and July–October intervals were used instead of shell height, to compare among stocks and between sites. The December–April and April–July interval growth rates among stocks and between sites were compared using two-factor ANOVA tests. The July–October interval growth rate among stocks was compared using a one-factor ANOVA test (4 GB stocks +3 MB stocks) as no PC oysters were left in Mobile Bay after July. The CI_DW_ and *P. marinus* infection intensity data in April and July were compared among stocks and between sites with two-factor ANOVA tests. The same data collected in October were compared with a one-factor ANOVA test (4 GB stocks +3 MB stocks). Shell height and *P. marinus* infection intensity data were log_10_-transformed to achieve normality and homogeneity of variance. All ANOVA tests were followed by Tukey’s multiple comparison tests as appropriate when significant differences (*P* < 0.05) were found. All statistical analyses were performed using R 3.6.0 (R Foundation for Statistical Computing, 2019).

### Salinity tolerance following acclimation under controlled laboratory conditions

#### Experimental design and methods

In mid-November 2018, oysters (~3 months old) from each stock were transferred from Bayou Sullivan (salinity, 24.0; 12.3°C) to the static systems at LSU AFL. A subset of 15 oysters from each stock was haphazardly sampled to measure shell height and determine initial CI_DW_. Initial mean shell height and CI (± SD) for each stock were as follows: PC, 28.6 ± 2.9 mm, 5.9 ± 0.7; AB, 27.5 ± 4.7 mm, 7.7 ± 1.1; CL, 22.9 ± 2.9 mm, 6.6 ± 1.3; and VB, 25.8 ± 2.2, 7.3 ± 2.0. Fifty oysters of each stock were placed in ten 400-l tanks filled with aerated artificial seawater (Crystal Sea Marinemix, Marine Enterprises International, Baltimore, Maryland) with a salinity of 20.0 and maintained at a temperature of 20.0°C.

Salinity in each tank was gradually adjusted at a rate of 3 units every 2–3 days until the target salinities of 2.0, 4.0, 22.0, 38.0 and 44.0 were reached (i.e. after 16, 14, 3, 17 and 22 days, respectively). The treatments were replicated twice (5 salinity treatments × 2 replicate groups = 10 tanks). Temperature was also gradually adjusted at a rate of 1.0°C every 2 days until the experimental temperature of 25°C was reached (i.e. after 10 days). Oysters were fed daily at ~5% of their dry meat weight with Shellfish Diet 1800® (Reed Mariculture Inc., Campbell, California) when visual observation indicated that all algae added the day before had been cleared from the water. Every other day, the number of live and dead oysters was counted and dead oysters were removed from each tank. Oysters were considered dead when they were unable to effect shell closure when squeezed at least five times. Cumulative mortalities were calculated as described by Ragone Calvo *et al.* (2003) until the end to the experiment in February 2019 (11 weeks). Salinity and temperature were checked every other day and adjusted as needed.

After 1 month in the tanks, hemolymph was collected from three oysters per stock at each salinity (1 or 2 oysters per tank) and centrifuged at 400 × *g* for 10 min (3 oysters × 4 stocks × 5 salinities = 60 samples). The oysters were not replaced. The supernatant, or plasma, was collected and its osmolality measured twice with a vapor pressure osmometer (Wescor Inc., Logan, Utah). At the end of the study, the ash-free dry weight (AFDW)-based CI (CI_AFDW_) of 15 oysters of each stock at each salinity was determined by dividing the AFDW by the weight of the whole wet oyster minus its shell wet weight and multiplying by 100 using a modification of the formula of Abbe and Albright (2003). The AFDW was used to eliminate the contribution of salts so that the CI could be compared among oysters across the broad range of salinities used.

#### Statistical analyses

We tested for effects of stock, salinity and number of days of exposure on mortality via logistic regression. We constructed all possible candidate models from combination of the three predictor variables and their interaction terms and then tested these candidate models using Akaike’s information criterion AICc (Burnham and Anderson, 1998). We considered the model with the minimum AICc value, and any model within two AIC units of the minimum value to be supported.

Differences in cumulative mortality (%) among stocks within each salinity at the end of the study were determined using a series of chi-square analyses with Bonferroni correction. The osmolalities of plasma collected from each stock after one month were compared using a one-factor ANOVA test for each salinity treatment. CI_AFDW_ at the end of the experiment were compared using a two-factor (stock and salinity treatment) ANOVA test, followed by Tukey’s multiple comparison test.

### Salinity tolerance without acclimation under controlled laboratory conditions

#### Experimental design and methods

The salinity tolerance experiment without acclimation was conducted in November–December 2018 (Trial 1) and March–April 2019 (Trial 2) at Texas A&M University-Corpus Christi using oysters transferred from Bayou Sullivan. Salinities and temperatures at the time of collection at Bayou Sullivan were 24.0 and 12.3°C in November and 14.5 and 19.7°C in March. The mean shell heights (± SD, *N* = 25) for each stock in November 2018 were as follows: PC, 29.4 ± 3.8; AB, 26.4 ± 3.5; CL, 23.6 ± 2.9; and VB, 26.0 ± 3.6 mm. The mean shell heights for each stock in March 2019 were as follows: PC, 33.1 ± 4.8; AB, 35.9 ± 4.2; CL, 38.4 ± 5.6; and VB, 37.0 ± 4.4.

In each trial, 25 oysters from each stock were placed in fifteen 38-l tanks (100 oysters per tank) with aerated artificial seawater (using Instant Ocean Reef Crystals Reef Salt, Blacksburg, Virginia) at 25°C and salinities of 2.0, 4.0, 20.0, 38.0 and 44.0; each salinity treatment was replicated three times (5 salinity treatments × 3 replicate groups = 15 tanks). Temperature and salinity from each tank were recorded daily. Oysters were fed daily with Shellfish Diet 1800® as in the previous laboratory study. Every other day, the numbers of live and dead oysters of each stock in each tank were counted over a 3-week period and the dead oysters were removed.

#### Statistical analyses

Mortality data were compared among stocks and salinity treatments using probit analysis (R package ‘*ecotox*’, Wheeler *et al.,* 2006). Median lethal time (LT_50_) with 95% confidence intervals was determined for each stock and salinity treatment. LT_50_ are considered statistically different when confidence intervals do not overlap.

**Figure 2:**
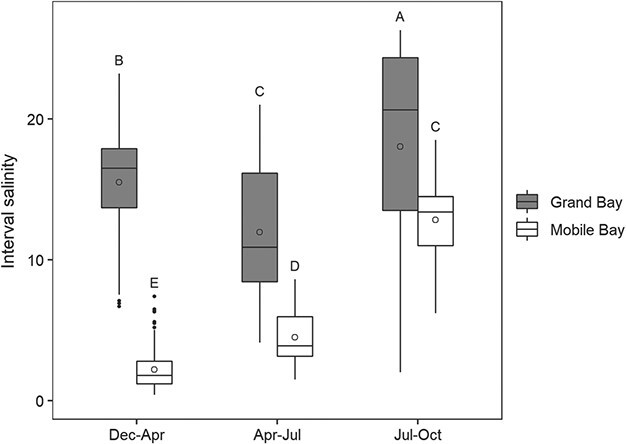
Salinity at the Alabama Grand Bay and Mobile Bay sites for the December 2018–April 2019, April–July and July–October intervals. The boundaries of the box represent the 25th and 75th percentiles, while the line within the box is the median. The mean is represented with a circle. Error bars indicate 1.5*IQR above and below the box boundaries, respectively. Groups with different letters are statistically different (*P* < 0.05).

## Results

### Salinity tolerance during field exposure

#### Salinity and temperature

Monthly mean salinity in Mobile Bay ranged from 1.5 ± 0.6 in January 2019 to 14.4 ± 1.8 in October, with a daily minimum of 0.4 in March and a daily maximum of 18.5 in July (Supplemental Fig. 3). Monthly mean salinity (± SD) for Grand Bay at PAC ranged from 9.0 ± 2.4 in May to 25.0 ± 1.0 in September with a daily minimum of 2.0 in July and a daily maximum of 26.4 in December (Supplemental Fig. 3).

There was a significant site–interval interaction for salinity (*P* < 0.001). Overall, salinities were significantly lower at the Mobile Bay site (December–April, 2.2 ± 1.5; April–July, 4.5 ± 1.9; July–October, 12.8 ± 2.9) than at the Grand Bay site (December–April, 15.5 ± 3.6; April–July, 12.0 ± 4.8; July–October, 18.0 ± 7.9) for each time interval (Fig. 2, Supplemental Table 3).

Temperature followed seasonal patterns with monthly means ranging from 14.4 ± 2.4°C in Grand Bay and 12.9 ± 3.2°C in Mobile Bay in January to 30.3 ± 1.1°C in July in Grand Bay and 29.7 ± 1.3°C in Mobile Bay in August (Supplemental Fig. 3).

Temperature was significantly affected by interval (*P* < 0.001) and site (*P* < 0.001). Temperature increased from December–April (15.4 ± 3.4°C) to April–July (26.7 ± 3.4°C) and again from April–July to July–October (29.8 ± 1.4°C) intervals and was significantly greater at the Grand Bay site (24.4 ± 6.6°C) than at Mobile Bay site (22.7 ± 7.2°C) (Supplemental Table 3).

#### Mortality

**Figure 3:**
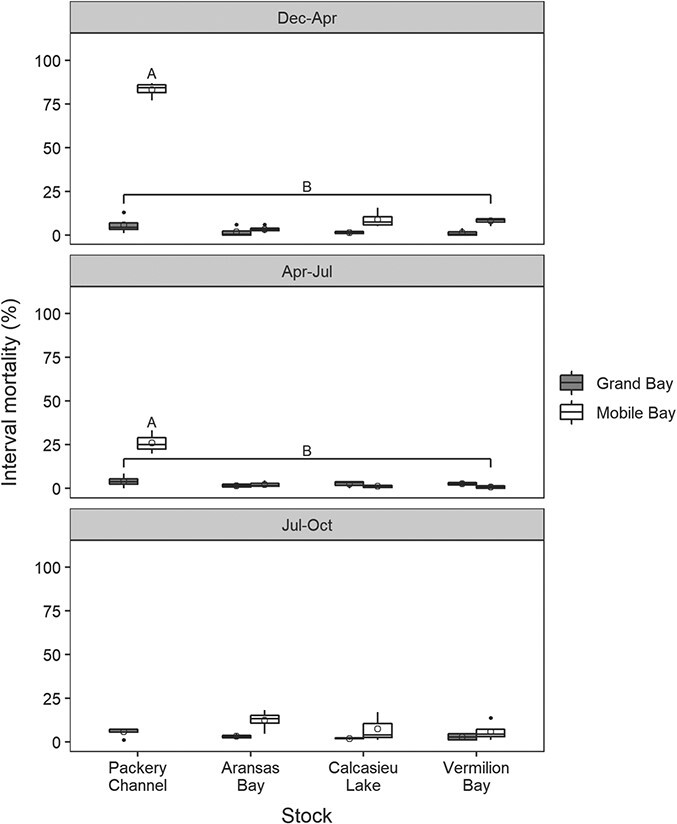
Interval mortality for the progenies of the four oyster broodstocks at the Alabama Grand Bay and Mobile Bay sites for the December 2018–April 2019, April–July and July–October intervals. Box features are described in Fig. 2. Groups with different letters are statistically different (*P* < 0.05).

There was a significant (*P* < 0.001) stock–site interaction in oyster interval mortality for December–April. The interval mortality of all stocks at the Grand Bay site was significantly less (*P* < 0.001) than all stocks at the Mobile Bay site (Fig. 3). Moreover, the interval mortality of PC oysters (83.1 ± 4.3%) at the Mobile Bay site was significantly greater than that of the other stocks (all <10%) at either site.

Similar to the Decemeber–April analysis, there was a significant (*P* < 0.001) stock–site interaction in the April–July interval mortality, with PC oysters (26.1 ± 6.7%) at Mobile Bay having significantly greater mortality than all other stocks (all < 5%) at either site.

For the July–October interval, there were no significant differences in interval mortality between stocks (4 GB + 3 MB; *P* = 0.063, one-factor ANOVA), and all stocks maintained mortalities of <13%. Monthly changes in cumulative mortality for each stock at each site are shown in Supplemental Fig. 4.

#### Initial shell height and growth rate

There was a significant (*P* < 0.001) stock–site interaction in the initial shell height of oysters at the time of deployment. PC oysters deployed at the Mobile Bay (33.6 ± 4.5 mm) and Grand Bay (32.6 ± 4.7 mm) sites were significantly larger than all other stocks (*P* < 0.001, Supplementary Table 1). CL oysters at the Mobile Bay (26.3 ± 3.0 mm) and Grand Bay (27.5 ± 3.3 mm) sites were significantly smaller than all other stocks except at the Grand Bay site where no difference between CL and AB oysters could be shown. Monthly changes in shell heights for each stock at each site are shown in Supplemental Fig. 5.

**Figure 4:**
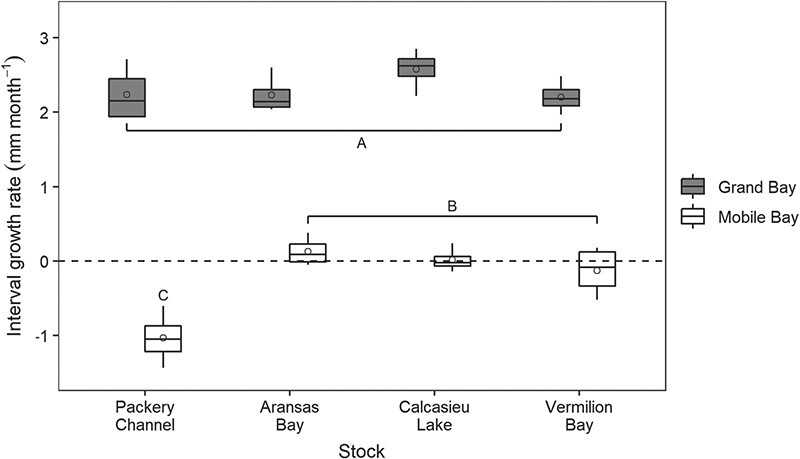
Interval growth rate for the progenies of the four oyster broodstocks at the Alabama Grand Bay and Mobile Bay sites between December 2018 and April 2019. Box features are described in Fig. 2. Groups with different letters are statistically different (*P* < 0.05).

There was a significant (*P* = 0.002) stock–site interaction in oyster interval growth rate for December–April. The growth rates of all stocks at the Mobile Bay site were significantly less (*P* < 0.001) than all stocks at the Grand Bay site (Fig. 4). In addition, at the Mobile Bay site, the interval growth rate of PC oysters was significantly lower than that of the other stocks and negative, indicating that for PC oysters more of the larger oysters died than smaller ones between December and April (Fig. 4).

For the April–July interval, growth rate significantly differed between sites (*P* = 0.016) with growth rate at the Grand Bay site (4.0 ± 0.9 mm month^−1^) being significantly greater than at the Mobile Bay site (2.7 ± 0.8 mm month^−1^).

For the July–October one-factor ANOVA test (4 GB + 3 MB), only the PC stock from the Grand Bay site (6.7 ± 0.6 mm month^−1^) was significantly greater than the AB stock from Mobile Bay (5.0 ± 0.6 mm month^−1^). Interval growth rates for each stock at each site are provided in Supplemental Table 3.

#### Condition index

The CI_DW_ in April was significantly affected by site (*P* < 0.001) with significantly greater CI_DW_ at the Mobile Bay site (14.7 ± 3.0) than at the Grand Bay site (13.4 ± 1.8) (Supplemental Fig. 6).

In July, the CI_DW_ was significantly affected by site (*P* < 0.001) and stock (*P* < 0.001). The CI_DW_ continued to be significantly greater at the Mobile Bay site (13.5 ± 1.5) than at the Grand Bay Site (9.0 ± 1.4). In addition, the CI_DW_ of the PC stock (9.8 ± 2.4) was significantly less than all other stocks (AB: 11.3 ± 2.6, *P* = 0.005; CL: 11.6 ± 3.0, *P* < 0.001; VB: 11.7 ± 2.3, *P* < 0.001).

In October, the one-factor ANOVA test (4 GB + 3 MB) indicated the CI_DW_ of all stocks at the Mobile Bay site (AB_MB_: 9.57 ± 0.64, CL_MB_: 9.67 ± 0.86, VB_MB_: 9.48 ± 2.04) were significantly greater than those of all stocks at the Grand Bay site (PC_GB_: 6.63 ± 1.21, AB_GB_: 7.83 ± 0.97, CL_GB_: 8.01 ± 1.24, VB_GB_: 7.69 ± 1.26). Additionally, the CI_DW_ of PC_GB_ was significantly less than that of AB_GB_ and CL_GB_ (*P* = 0.044 and *P* = 0.011, respectively), but not VB_GB_ (*P* = 0.111)_._

#### *Perkinsus marinus* infection intensity

*Perkinsus marinus* infection intensity in April was significantly affected by site (*P* < 0.001) and stock (*P* = 0.017) (Supplemental Fig. 7). The *P. marinus* infection intensity (log_10_ parasites g^−1^ wet tissue) was significantly greater at the Grand Bay site (2.79 ± 0.51) than the Mobile Bay site (2.05 ± 0.75) and was significantly greater (*P* = 0.013) in CL oysters (2.66 ± 0.42) than in VB oysters (2.24 ± 0.86).

In July, the infection intensity was significantly affected by site (*P* < 0.001) with significantly greater infection intensity at the Grand Bay site (2.27 ± 0.57) than at the Mobile Bay site (1.51 ± 0.83).

In October, the one-factor ANOVA test (4 GB + 3 MB) indicated significant differences in *P. marinus* infection intensity among stocks (*P* = 0.008), but no significant differences between stocks could be found using Tukey’s Multiple Comparison of Means.

### Salinity tolerance following acclimation

#### Plasma osmolality

Osmolality ranged from 80 ± 5 mOsm kg^−1^ (salinity 2.0, CL) to 1340 ± 4 mOsm kg^−1^ (salinity 44.0, PC), with osmolality increasing with salinity (Table 2). There were no significant differences in osmolality among stocks at each salinity (*P* > 0.1), except for marginally lower osmolalities for VB (1147 ± 1 mOsm kg^−1^) versus PC (1153 ± 2 mOsm kg^−1^) stocks at salinity 38 (*P* = 0.03).

**Table 2:** Mean plasma osmolality (mOsm kg^−1^, ± SD) of the progenies of Texas oysters from PC and AB broodstocks and of Louisiana oysters from CL and VB broodstocks, 1 month after the start of the study to determine the salinity tolerance following acclimation at salinities of 2.0, 4.0, 22.0, 38.0 or 44.0

Salinity/osmolality (mOsm kg^−1^) equivalence															
Stock	2.0/71			4.0/130			22.0/678			38.0/1142			44.0/1326		
PC	112.00	±	38.50	140.00	±	3.61	675.67	±	4.51	1153.67	±	2.08	1339.67	±	3.79
AB	80.67	±	1.53	138.33	±	4.04	680.00	±	6.56	1149.67	±	3.06	1334.67	±	10.02
CL	80.67	±	4.73	136.33	±	0.58	676.33	±	1.53	1150.33	±	2.08	1339.33	±	10.02
VB	89.00	±	14.21	134.67	±	2.89	684.00	±	1.00	1146.67	±	1.15	1331.67	±	3.06

#### Mortality

Under both low (2, 4, 20) and high (20, 36, 44) salinity, the best supported logistic regression models all included effects of stock, salinity and number of days of exposure on mortality (Supplemental Table 4).

**Figure 5:**
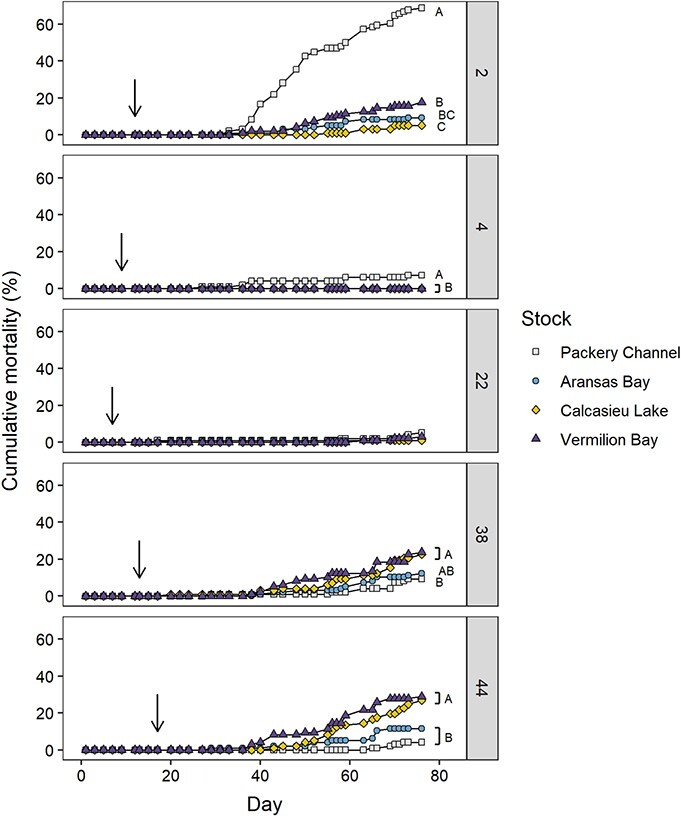
Cumulative mortality of the progenies of the four oyster broodstocks at salinities of 2.0, 4.0, 22.0, 38.0 and 44.0 following salinity acclimation. Arrows indicate the day at which each treatment reached its target salinity. Groups with different letters are statistically different (*P* < 0.05) at each salinity.

At the end of the experiment, significant differences in cumulative mortality were found among stocks at salinity 2.0 (*P* < 0.001), 4.0 (*P* < 0.001), 38.0 (*P* = 0.026) and 44.0 (*P* < 0.001; Fig. 5). At salinity 2.0, the cumulative mortality of PC oysters (68.8%) was significantly higher than AB, CL and VB (9.3%, 5.2% and 17.7%, respectively; *P* < 0.001). At salinity 4.0, the cumulative mortality of PC oysters (7.3%) was significantly higher than all other stocks (*P* = 0.042), which experienced no mortality. There were no significant differences in mortality among stocks at salinity 22.0 (*P* = 0.187). At salinity 38.0, Louisiana stocks (CL, 22.7%; VB, 23.7%) had significantly higher cumulative mortality than PC (9.3%; *P* = 0.018 and *P* = 0.012, respectively), but were not different from AB (12.4%; *P* = 0.082 and *P* = 0.059, respectively). At salinity 44.0, cumulative mortality of Louisiana stocks (CL, 26.8%; VB, 28.9%) was significantly higher than the PC stock (4.1%; *P* < 0.0001), and the VB stock was significantly higher than the AB stock (11.4%; *P* = 0.036).

#### Condition index

**Figure 6:**
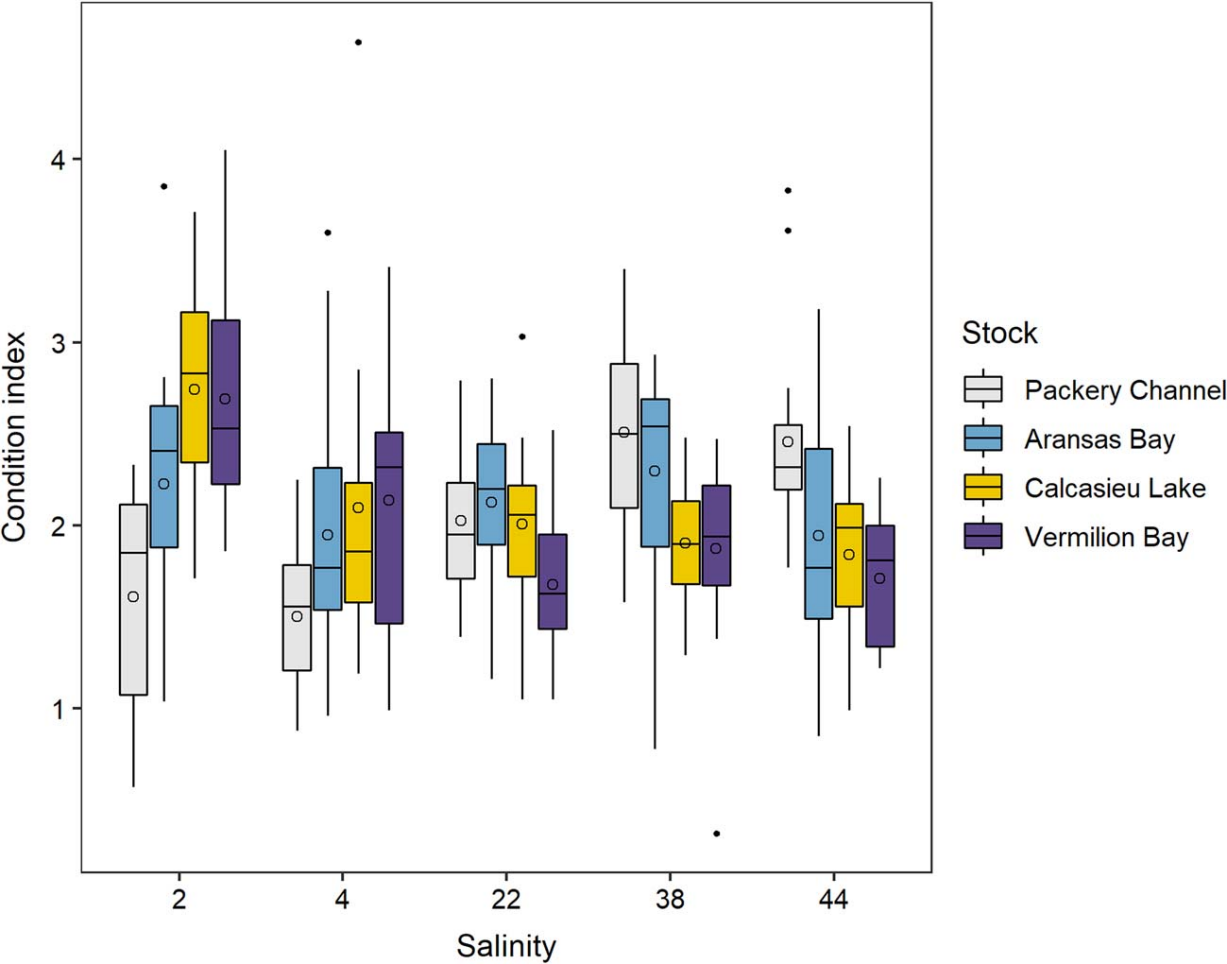
CI of the progenies of the four oyster broodstocks at salinities of 2.0, 4.0, 22.0, 38.0 or 44.0 in the study with acclimation. Box features are described in Fig. 3.

There was a significant (*P* < 0.001) stock–salinity treatment interaction for CI_AFDW_ at the end of the experiment. In general, the CIs of Louisiana stocks were higher at the lower salinity treatments, while CI_AFDW_ of Texas stocks were higher (PC) or tended to be higher (AB) at the higher salinity treatments at the end of the experiment (Fig. 6). Specifically, the CI_AFDW_ of CL at salinity 2.0 (2.74 ± 0.58) was significantly higher than at 38.0 (1.90 ± 0.36; *P* = 0.013) and 44.0 (1.84 ± 0.41; *P* = 0.004). Similarly, the CI_AFDW_ of VB at salinity 2.0 (2.69 ± 0.66) was significantly higher than at 22.0 (1.68 ± 0.42; *P* < 0.001), 38.0 (1.90 ± 0.36; *P* = 0.019) and 44.0 (1.71 ± 0.37; *P* < 0.001). In contrast, the CI_AFDW_ of PC at salinity 2.0 (1.61 ± 0.64) was significantly lower than at salinities of 38.0 (2.51 ± 0.55; *P* = 0.005) and 44.0 (2.46 ± 0.58; *P* = 0.011).

Significant differences in CI_AFDW_ were also found among stocks (Fig. 6). Specifically, CI_AFDW_ of CL at salinity 2.0 was significantly higher than CI_AFDW_ of PC at salinity 2.0 (*P* < 0.001) and CI_AFDW_ of PC and AB at salinity 4.0 (1.50 ± 0.39, *P* < 0.001 and 1.95 ± 0.74, *P* = 0.027, respectfully). Similarly, CI_AFDW_ of VB at salinity 2.0 was significantly higher than the CI of PC at salinity 2.0 and 4.0 (*P* < 0.001). At the higher salinities, only the CI_AFDW_ of PC at salinity 38.0 (2.51 ± 0.55) was significantly higher than CI_AFDW_ of VB at salinity 22.0 (1.68 ± 0.42; *P* = 0.015) and 44.0 (1.71 ± 0.37; *P* = 0.027). However, the CI_AFDW_ of Texas oysters tended to be higher than the CI_AFDW_ of Louisiana oysters.

### Salinity tolerance without acclimation

#### Mortality

**Figure 7:**
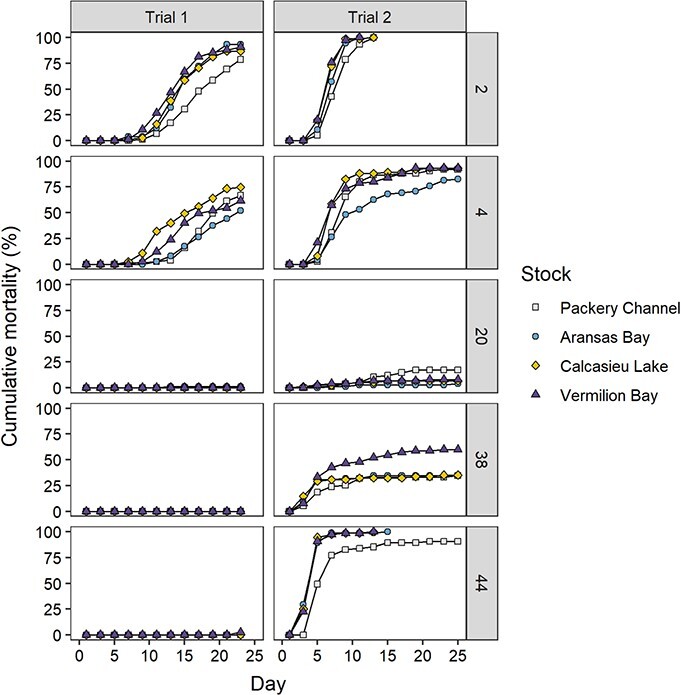
Cumulative mortality of the progenies of the four oyster broodstocks at salinities of 2.0, 4.0, 20.0, 38.0 or 44.0 in experiments without salinity acclimation. Trial 1 was done with 3-month-old oysters (mean stock SH range, 23.6–29.34 mm) collected at a field salinity of 24, while 7-month-old oysters (33.1–38.4 mm) collected at a field salinity of 14.5 were used for Trial 2.

Mortality of oysters from each stock exposed to treatment salinities differed between Trials 1 and 2. At the end of Trial 1 (23 days), mortalities of oysters exposed to salinity 2.0 and 4.0 were high (ranging from 78–93% to 52–75%, respectively), whereas mortalities of oysters exposed to salinity 20.0, 38.0 and 44.0 were low (<1%, 0 and <3%, respectively; Fig. 7). In Trial 1, significant differences in LT_50_ were found among stocks (Table 3) at salinity 2.0 and 4.0. At salinity 2.0, PC stock had significantly higher LT_50_ (lower mortality, 17.9) than all other stocks (AB, 14.8; CL, 14.9; VB, 13.8). At salinity 4.0, CL stock had significantly lower LT_50_ (higher mortality, 16.0) than all other stocks (PC, 19.8; AB, 21.4; VB, 18.7). In Trial 1, LT_50_ values could not be calculated for any stocks exposed to salinities of 20 or higher because mortality rates were too low (<3%).

**Table 3:** Median lethal tolerance (LT_50_) and 95% confidence interval (95% Conf. Int.) results of the probit analysis for each salinity and stock during Trials 1 and 2 of the study without acclimation

		Trial 1 (23 days)			Trial 2 (25 days)		
Salinity	Stock	Slope	LT_50_	95% Conf. Int	Slope	LT_50_	95% Conf. Int.
2	PC	0.2150	17.9	(17.2, 18.8)	0.5810	7.6	(7.19, 7.97)
	AB	0.2690	14.8	(14.2, 15.5)	0.7360	6.8	(6.44, 7.09)
	CL	0.2570	14.9	(14.3, 15.7)	0.7740	6.2	(5.90, 6.52)
	VB	0.2600	13.8	(13.0, 14.9)	0.7980	6.1	(5.83, 6.44)
4	PC	0.2080	19.8	(19.0, 20.8)	0.2460	9.8	
	AB	0.1740	21.4	(20.4, 22.8)	0.1430	13.1	(11.0, 16.1)
	CL	0.1710	16.0	(14.7, 17.7)	0.2740	8.3	
	VB	0.1640	18.7	(17.2, 20.6)	0.2280	8.6	
20	PC				0.0684	34.9	(30.6, 42.2)
	AB						
	CL						
	VB						
38	PC				0.0453	28.2	(22.1, 45.3)
	AB				0.0361	29.3	(21.3, 70.1)
	CL				0.0334	30.7	(22.1, 73.0)
	VB				0.0711	15.4	(12.0, 21.2)
44	PC				0.1990	7.5	
	AB				0.9250	3.6	
	CL				1.0900	3.6	
	VB				1.0000	3.8	

Treatments with no LT_50_ reported had a cumulative mortality of <10%; treatments with insufficient data to estimate 95% confidence intervals have no confidence intervals reported.

LT_50_ are considered statistically different when confidence intervals do not overlap.

Trial 1 was done with 3-month-old oysters (mean stock SH range, 23.6–29.34 mm) collected at a field salinity of 24, while 7-month-old oysters (33.1–38.4 mm) collected at a field salinity of 14.5 were used for Trial 2.

At the end of Trial 2 (25 days), mortality was greatest at the highest and lowest salinities (salinity 2.0: 100%, 44.0: range of 90–100%), followed by the more moderate salinities tested (salinity 4.0: 82–93%, 38.0: 34–60%), with lowest mortalities at salinity 20.0 (0–17%). Greater than 50% mortality was achieved in 5 days for all stocks at salinity 44.0, and in 1 week for the three lower salinity stocks (VB, CL, AB) at salinity 2.0 and for the two lowest salinity stocks (VB, CL) at salinity 4.0 (Fig. 7). Significant differences in LT_50_ were found among stocks in Trial 2 (Table 3). At salinity 2.0, PC had significantly higher LT_50_ (lower mortality, 7.6) than all other stocks (AB, 6.8; CL, 6.2; VB, 6.1). At salinity 4.0, AB had lower mortality than all other stocks, but 95% confidence intervals could not be calculated for the other stocks. No LT_50_ values could be calculated for any stock at salinity 20.0. At salinity 38.0, VB had a significantly lower LT_50_ (15.4) than all other stocks (PC, 28.2; AB, 29.3; CL, 30.7). At salinity 44.0, the mortalities went from 0% on Day 1 to >89% on Day 5 for AB, CL and VB stocks or >75% on Day 7 for PC stock. The LT_50_ of the AB (3.6), CL (3.6) and VB (3.6) stocks seemed lower than that of the PC stock (7.5) but no 95% confidence intervals could be obtained using Probit analysis because too few data points were collected (i.e. every 2 days) when most mortalities occurred.

## Discussion

Using a combination of laboratory and field studies on F1 cohorts, we tested whether GoM populations of a key estuarine foundation species differ in their physiological responses to salinity. Salinity tolerance varied among GoM oyster stocks acclimated to extreme salinities in the laboratory. The Texas stock originating from the highest salinity estuary (PC) performed significantly worse at lower salinities (2.0, 4.0) and better at higher salinities (38.0, 44.0) than the Louisiana stocks from the lower salinity estuaries (CL, VB). The Texas stock from a moderate salinity estuary (AB) performed as well as the PC stock at high salinity and as well as the Louisiana stocks at low salinity. Field results corroborated these laboratory findings, providing evidence of local adaptation to salinity. Different laboratory results were obtained, however, when the F1 oysters were transferred directly, without acclimation, to extreme salinities.

The PC stock experienced, by far, the greatest mortality at low salinities (≤ 4), whether following acclimation in the laboratory or in the field. PC oysters started dying after about a month at extreme low salinity and over 50% were dead within another month in contrast to the other stocks that had mortality of <5% in the field and 15% in the laboratory. It is clear that PC oysters were not as competent as the other stocks in handling low salinity. During the laboratory acclimation study, the mean plasma osmolalities of all stocks were >80 mOsm kg^−1^ and constantly hyperosmotic to the seawater at a salinity of 2 (~71 mOsm kg^−1^ equivalent) with PC stock more so than the other stocks. PC stock plasma osmolality was also more variable at a salinity of 2.0 indicating more differences among PC oysters in their ability to osmoconform at that extreme salinity. This is in agreement with past studies that found a minimum plasma osmolality limit of about 80 mOsm kg^−1^ (salinity of ~2.4 equivalent) in Louisiana oysters at a low salinity field site (Cow Bayou, Louisiana) or exposed to simulated freshet events (< 3) (La Peyre *et al.,* 2003, 2009). The difference in osmolarity between plasma and the diluted seawater is more than would be expected as hemolymph of oysters has been found to be only slightly hyperosmotic (<10 mOsm kg^−1^) in this osmoconformer (Hand and Stickle, 1977; Shumway, 1977). This hypotonic challenge would result in excessive water movement into cells and cells having to move water out to keep cell volume within tolerable range, along with salt loss, when oyster valves are opened. Closing their valves allow oysters to shield their epithelial cells from the uptake of water when under hypoosmotic exposure but requires oysters to switch to anaerobic metabolism, which is less energy efficient. Oysters at low salinity (3) do stay closed far longer than at higher salinities and feeding practically ceases limiting any energy acquisition (Casas *et al.,* 2018). Preliminary measurements of valve movement indicate that oysters do open occasionally but only for short periods at very low salinity, likely to eliminate metabolites (J La Peyre, unpublished). In either case, a net energy must be expended by the oysters to survive at this low salinity. The lower CI of the PC stock compared to the VB and CL stocks acclimated to low salinity in the laboratory suggest PC oysters spent more energy in counteracting the effects of low salinity which likely lead, eventually, to ATP being depleted sooner followed by death (Sokolova *et al.,* 2012; Fuhrmann *et al.,* 2018).

In contrast to the results at low salinity, the PC stock was more tolerant to the highest salinities than the CL and VB stocks following acclimation in the laboratory, although differences were much less dramatic than at the low salinities. The mortality of PC oysters was significantly less and the mean CI was significantly greater than those of Louisiana oysters*.* In this case, PC oysters were better able to cope with higher salinities than the other stocks. The underlying cellular, biochemical and molecular mechanisms for the physiological differences in the contrasting salinity tolerance between stocks are unknown and will need to be determined in future studies. Interestingly, a past study identified differences in both tissue concentration and composition of the amino acid pools, used as osmolytes, of oysters between Chesapeake Bay and Atlantic oysters, along the mid-Atlantic United States coast (Pierce *et al.,* 1992). Atlantic oysters primarily used taurine while Chesapeake Bay oysters, which are exposed to lower salinity than Atlantic oysters, relied on alanine, glycine and proline to acclimate to high salinity. In addition, glycine betaine, a quaternary amine, is an important osmolyte for cell volume regulation in response to salinity changes in Atlantic oysters but not Chesapeake Bay oysters (Pierce *et al.,* 1992; Pierce *et al.,* 1995). Whether or not similar differences in organic osmolytes use are found between the PC and other stock oysters remain to be determined. Variation in ionic content and cell volume regulation with salinity changes among stocks will need to be compared along with other potential differences in the cellular mechanisms involved in adaptation to salinity changes such as reversible changes in protein and RNA synthesis and alteration of the pattern of multiple molecular forms of different enzymes (Pierce, 1982; Berger and Kharazova, 1997).

The findings that the PC stock outperformed VB and CL stocks at high salinities and did poorly at low salinity is not surprising because PC oysters were collected in the Upper Laguna Madre, a hypersaline estuary, where oysters are predominantly exposed to high salinity and seldom to low salinity events, while VB and CL oysters are exposed to lower salinities with increasing frequencies in Louisiana (Breuer, 1962; Soniat *et al.,* 2013; Wang *et al.,* 2017), and rarely to high salinities. Under such persistent differences in salinity and geographic semi-solation, adaptation across generations would be expected (King *et al.,* 1994; Sanford and Kelly, 2011; Anderson *et al.,* 2014; Maynard *et al.,* 2018). In fact, there is strong evidence of genetic differentiation between Laguna Madre and other GoM oyster populations (Groue and Lester, 1982; Buroker, 1983; King *et al.,* 1994; Varney *et al.,* 2009; Anderson *et al.,* 2014) but with increasing migration of upper Laguna Madre oysters eastward and some limited hybridization with Corpus Christi/Aransas Bay oyster populations following hydrological changes associated with the 1949 opening of the Gulf Intracoastal Waterway (Anderson *et al.,* 2014). Buroker (1983) already suggested some populations may be unable to adapt to certain environmental conditions, such as the hypersaline conditions of the Laguna Madre or the variability in freshwater inflow observed in the Mississippi delta estuaries, causing genotypes in some populations to be favored over others. Our physiological studies characterizing responses to extreme salinities, provide evidence that PC oysters have phenotypes, which improve survival in high-salinity conditions and are maladapted (i.e. experience extreme mortalities) in low-salinity conditions. Potential intergenerational or within generation carryover effects on the performance of our oysters also need to be recognized. Parental exposure can affect offspring phenotypes and early life exposure can affect the performance of oysters later in life without changes of DNA sequence (Spencer *et al.,* 2020; Donelan *et al.,* 2021). Maintaining wild Louisiana broodstocks collected from CL and VB at Grand Isle, a site with higher mean salinity (and possibly different in other environmental conditions) to ensure gonad development prior to spawning, may have impacted the performance of their progeny through changes in DNA methylation. The Texas wild broodstocks, in contrast, went through gametogenesis at their home conditions and were not acclimatized before spawning. It has been shown that epigenetic divergence exceeds genetic divergence in some Louisiana oyster populations (Johnson and Kelly, 2020). However, another study found no evidence for parental carryover effects on larval tolerance of low-salinity conditions, even after parental stocks were maintained at low-salinity conditions for 2 years (Griffiths *et al.,* 2021). While our studies were limited to characterizing the performance of the progeny of wild oysters (i.e. F1) producing subsequent generations of oysters would help elucidate the specific contribution of the genome versus the epigenome in oyster salinity tolerance (Kawecki and Ebert, 2004).

Low mortality of Louisiana stocks at low salinities has been noted in previous studies (Casas *et al.,* 2017; Leonhardt *et al.,* 2017; Lowe *et al.,* 2017), especially when temperatures are not exceedingly high (<30°C, Rybovich *et al.,* 2016). One recent study compared Alabama oyster stocks at four sites, with results indicating high mortality in Mobile Bay concomitant with prolonged low salinity (<5 for 45 days) when temperatures were consistently >28°C in July and August 2016 (Wadsworth *et al.,* 2019). In contrast, in our current study, AB, CL and VB stocks experienced overall low mortality in Mobile Bay, but the duration of exposure to salinity < 5 when temperatures were >28°C was only 19 days in late May through early June 2019. Laboratory results also indicate limited mortality (<5%) of oysters from several Louisiana stocks maintained at a salinity of 3 for more than 3 months at 25°C (J La Peyre, unpublished data)*.* Similarly, Butler (1952) reported self-sustaining oyster populations in areas of the Mississippi River delta with salinities below 3.5 for five consecutive months. Further studies are needed to determine whether or not there are more subtle (than PC versus AB, VB, CL) but still significant differences in low salinity tolerance among oysters from the varied estuaries of the GoM.

While many studies have reported the effects of low salinity and freshet events on oysters, recent efforts have focused on differences in survival and growth among oyster populations at low salinity and under freshet events in view of their predicted increase in frequency with climate change (Eierman and Hare, 2013; Méthé *et al.,* 2015; Bible and Sanford, 2016; Leonhardt *et al.,* 2017; Scharping *et al.,* 2019; McCarty *et al.,* 2020)*.* Studies relating genomic variation, including in osmoregulation-related genes, with salinity tolerance may enable the development of biomarkers that managers could use to identify stocks best suited to outplant environmental conditions for restoration efforts or aquaculture expansion. The identification of candidate genes for osmotic regulation and metabolic pathways used in osmoregulation in various oyster species may assist in investigating the genetics of the mechanisms underlying salinity tolerance in GoM oysters and in breeding oysters that are more tolerant to low-salinity considering the recently shown moderate heritability (h^2^ = 0.4) for low salinity survival in *C. virginica* (Zhang *et al.,* 2012; Meng *et al.,* 2013; Eierman and Hare, 2014, 2016; Maynard *et al.,* 2018; McCarty *et al.,* 2020). It is also important to note, however, that many other genes not specifically related to osmoregulation per se but responding to stress associated with changing salinity conditions might also play important roles in assuring survival (Zhang *et al.,* 2012; Meng *et al.,* 2013).

By the end of the field study, shell heights were greater among all stocks at Grand Bay compared with Mobile Bay, supporting previous studies showing faster growth rates at higher salinities (e.g. Bataller *et al.,* 1999; Livingston *et al.,* 2000; Kraeuter *et al.,* 2007; Wang *et al.,* 2008). The discrepancy in growth between the two sites is related to the prolonged low salinities in Mobile Bay following deployment. Besides a 3-week period in April where salinities were ≥4, salinities at the reference site were <4 from deployment through June, which corresponds with little to no shell growth in all stocks during that time. Low salinity significantly reduces oyster feeding while food quantity and quality is depressed (Riekenberg *et al.,* 2015; Lavaud *et al.,* 2017; Casas *et al.,* 2018). The negative growth rate of PC oysters between December and April at the Mobile Bay site also indicates that larger oysters died earlier than smaller ones in agreement with recent findings (Rybovich *et al.,* 2016; Southworth *et al.,* 2017). Decreased resilience of larger animals to low salinity and other environmental stressors has been reported in oysters and other animals and is attributed to size-related scaling effects on energetics (Widdows, 1978; Bayne and Newell, 1983; Sukhotin *et al.,* 2003; Yuan *et al.,* 2010; Munroe *et al.,* 2013; Rybovich *et al.,* 2016).

At both the low and intermediate salinity field sites, mean CI of all stocks gradually decreased from April to October, reflecting changes in gametogenic stages, the release of gametes via spawning and the associated increased metabolic rates (Supan and Wilson, 2001; Casas *et al.,* 2017; Leonhardt *et al.,* 2017; Casas *et al.,* 2018). Oyster spawning in the subtropical northern GoM can start as early as April, when water temperature and salinity exceed 25°C and 10, respectively, and last until October with gametogenic recycling (Hayes and Menzel, 1981; Supan and Wilson, 2001; Wadsworth *et al.,* 2019). Low water salinities (<10) are known to delay spawning, which explains why CI of stocks at Mobile Bay (salinity, <6 prior to July sampling) was greater than at Grand Bay (salinity, 9–17 prior to July sampling) in July and October (Butler, 1949; Loosanoff, 1953).

Most oysters sampled in our study were infected with *P. marinus*, which is endemic to GoM estuaries (Mackin, 1962; Craig *et al.,* 1989). Infection intensity was greater at the Grand Bay site than at the Mobile Bay site because of higher salinity, which promotes *P. marinus* proliferation (Chu *et al.,* 1993; Ragone Calvo *et al.,* 2003; La Peyre *et al.,* 2006). Nearly all oysters sampled throughout the study, however, had light infection intensities (<10^4^ parasites g^−1^ wet tissues) which is common in less than 1-year-old oysters and not expected to cause mortality (Bushek et al., 1994; La Peyre *et al.,* 2019b). There was little progression of the disease in most sampled oysters from April to October as salinity generally remained moderately low (<12) at both Mobile Bay and Grand Bay sites. Past field and laboratory studies have shown that lowered salinities (<12) delay *P. marinus* disease development (Chu *et al.,* 1993; Ragone Calvo *et al.,* 2003; Bushek *et al.,* 2012). Unfortunately, the overall low infection intensities and lack of disease progression during field deployment preclude any comparison of dermo resistance between stocks. Past studies have shown that the progeny of wild oysters from CL have a higher resistance to dermo than oysters from other Louisiana and Alabama estuaries (Casas *et al.,* 2017; Leonhardt *et al.,* 2017; La Peyre *et al.,* 2019b). The recent loss of over 90% of the CL oyster population (LDWF, 2018) due to overfishing and increased freshwater entering the estuary may reduce selection pressure for dermo resistance and illustrates how natural and anthropogenic variability can shift the multidirectional selection pressure oysters routinely face in estuarine environments. Considering that PC oysters grow in a fairly distinct high salinity environment, favorable conditions for *P. marinus* propagation, it will be important in future studies to determine whether or not they are potentially more resistant to dermo than other GoM stocks.

Mortalities differed between the two acute salinity tolerance trials. The major inconsistencies occurred at the highest salinities (38.0, 44.0) and were likely due to differences in the magnitude of salinity change F1 oysters were exposed to, from field collection to immersion, between trials. While most oysters in the first trial (collected at a field salinity of 24) survived direct immersion at salinities of 38.0 and 44.0, most oysters in the second trial (collected at a field salinity of 14.5) died when transferred to a salinity of 44, and between 25% and 50% died, depending on the stock, when transferred to a salinity of 38. It is also likely that oyster size and season contributed to some of the minor differences between trials. Oysters used in the first trial in November 2018 were 3-month-old juvenile oysters (mean stock SH range, 23.6–29.34 mm), compared to the 7-month-old oysters (33.1–38.4 mm) in the second trial in March 2019, which were larger and more mature with obvious gonad in development. Oysters that are larger and also closer to spawning have been reported to be more susceptible to stressors such as salinity and temperature changes and would explain the higher mortality rate (i.e. lower LT_50_) when exposed to extreme salinities than in the first trial (Huvet *et al.,* 2010; Rybovich *et al.,* 2016; Southworth *et al.,* 2017). Mortalities of the acute salinity tolerance trials also differed from the mortalities of the laboratory experiment of salinity tolerance following acclimation. In this case, the response of oysters acclimated to low salinity in the laboratory was the only good predictor of low salinity tolerance in the field.

Overall, direct transfer of F1 oysters into substantially different salinities caused rapid and greater mortalities indicating that oysters likely could not osmoconform fast enough and kept their valves essentially closed as a consequence. Oysters can take up to 4 weeks to fully osmoconform after transfer from seawater (salinity, 31–34) to 50% seawater (Heavers and Hammen, 1985). Differences in LT_50_ between stocks may reflect differences in the ability to withstand extended valve closures and survive anaerobically. Interestingly, the PC stock generally had higher LT_50_ times (i.e. required longer time to reach 50% mortality) than the other stocks upon transfer to extreme salinities. We also found in a preliminary study that PC oysters survived under hypoxic conditions (1 mg O_2_ L^−1^) longer than the other stocks (J La Peyre, unpublished data). This ability to survive longer under anaerobiosis might be related to the fact that oysters from Laguna Madre are predominantly exposed to changes in salinity at the higher end of estuarine salinity range, conditions under which amino acids plays a major role as osmolytes. It has been suggested that the synthesis of amino acids via known anaerobic biochemical pathways may facilitate high salinity acclimation (Baginski and Pierce, 1975; Henry *et al.,* 1980). Alternatively, PC oysters may simply be more resistant to acute stress regardless of the type of stressors.

It has long been assumed that local adaptation in marine environments was only evident in comparisons of populations on broad geographic scales due to dispersive larval or mobile adult stages (Conover *et al.,* 2006; Sanford and Kelly, 2011). This assumption was reinforced using early genetic methods with low resolution (e.g. allozyme frequencies) that are less variable and could identify only gross differences between populations. Buroker (1983), for example, only differentiated eastern oysters collected from Laguna Madre from oysters collected from other GoM and Atlantic estuaries. Using a higher resolution genetic marker (e.g. RFLP), eastern oysters could be further differentiated into North Atlantic, South Atlantic, east of Laguna Madre GoM and Laguna Madre populations (Reeb and Avise, 1990; Hoover and Gaffney, 2005; Varney *et al.,* 2009; Anderson *et al.,* 2014). More recent genotyping studies using single-nucleotide polymorphism (SNP) analyses are now able to detect more subtle differences in oyster populations that can be in close proximity (tens of km) (Eierman and Hare, 2016; Li *et al.,* 2018; Bernatchez *et al.,* 2019; Turley *et al.,* 2019; Johnson and Kelly, 2020). These SNP-related findings confirm the long-term view that oysters are potentially locally adapted because of observed distinct phenotypic variations when the progenies of oysters collected from different estuaries, were grown in the same environment (Barber *et al.,* 1991; Brown *et al.,* 1998; Dittman *et al.,* 1998; Burford *et al.,* 2014; Leonhardt *et al.,* 2017; Li *et al.,* 2018; Maynard *et al.,* 2018; La Peyre *et al.,* 2019b). In our study, PC oyster progenies had different physiological responses to salinity in both the field and laboratory studies compared with AB, CL and VB stocks. These data suggest adaptive divergence in the stock from PC, which is only 60 km from the AB broodstock site. Further investigation of GoM oyster populations under differing salinity regimes are needed to refine our understanding of the environmental distance or local adaptation between populations and the influence of genetic, maternal and epigenetic causes of phenotypic variation in stress tolerance.

In the US GoM, oyster production brings in >$100 M in average annual landings (2016–2018; NOAA, 2020). This production is supported by both wild oyster populations, resulting in significant investment in reef restoration, but also through the development of off-bottom aquaculture, with over 100 oyster farms established in the GoM region since 2009. Selecting locally adapted stocks could increase production. Productivity may be optimized by selecting stocks that are either locally adapted or have minimal ‘environmental distances’ between restored and local populations (McKay *et al.,* 2005; Bible and Sanford, 2016; Casas *et al.,* 2017; Leonhardt *et al.,* 2017). Likewise, consideration of anticipated changes in local and regional climate, climate change-related effects (e.g. sea level rise and ocean acidification), and anthropogenic changes (e.g. river or sediment diversions) in relation to stock performance is likely to increase production (Parker *et al.,* 2011; Wang *et al.,* 2017; Bernatchez *et al.,* 2019).

The results of these field and laboratory studies indicated that PC oysters (Upper Laguna Madre, Texas) are adapted to high-salinity conditions (>38) and do not tolerate very low salinities (<4). The AB stock (Texas) seemed to show the highest plasticity as it performed as well as the PC stock at the high salinities and as well as the Louisiana stocks at the lowest salinity. The AB stock also experiences more salinity variation from year to year as indicated by Supplemental Fig. 1. The Louisiana stocks did not perform as well as at the Texas stocks at high salinities. While *C. virginica* is a highly plastic species being tolerant to a broad range of salinity compared to many other oyster and bivalve species, significant differences in salinity tolerance between GoM stocks were found following sustained exposure to extreme salinities (≤2, ≥38). The differences in stock performance from this study not only highlight the importance of stock selection for aquaculture and restoration in estuaries that are currently, or will be in the future, experiencing extreme salinity conditions but also raise important questions about the potential genetic impact of hatchery-propagated oysters on wild populations.

## Funding

This work was supported by the National Science Foundation Biological Oceanography Program (grant number OCE 1731710) and Louisiana Sea Grant (award NA14OAR4170099).

## Supplementary Material

Supplemental_Material_coab065

## References

[ref1] Abbe GR, Albright BW (2003) An improvement to the determination of meat condition index for the eastern oyster *Crassostrea virginica* (Gmelin 1791). J Shellfish Res 22: 747–752.

[ref2] Anderson JD, Karel WJ, Mace CE, Bartram BL, Hare MP (2014) Spatial genetic features of eastern oysters (*Crassostrea virginica* Gmelin) in the Gulf of Mexico: northward movement of a secondary contact zone. Ecol Evol 4: 1671–1685. 24967084 10.1002/ece3.1064 PMC4063467

[ref3] Baginski RM, Pierce SK (1975) Anaerobiosis: a possible source of osmotic solute for high-salinity acclimation in marine molluscs. J Exp Biol 62: 589–598.

[ref4] Barber BJ, Ford SE, Wargo RN (1991) Genetic variation in the timing of gonadal maturation and spawning of the eastern oyster, *Crassostrea virginica* (Gmelin). Biol Bull 181: 216–221. 29304635 10.2307/1542092

[ref5] Bataller EE, Boghen AD, Burt MDB (1999) Comparative growth of the eastern oyster *Crassostrea virginica* (Gmelin) reared at low and high salinities in New Brunswick, Canada. J Shellfish Res 18: 107–114.

[ref6] Bayne B (2017) Biology of Oysters, Ed1 Vol 41. Academic Press, London

[ref7] Bayne BL, Newell RC (1983) Physiological energetics of marine molluscs. In ASM Saleuddin, KM Wilbur, eds, The Mollusca, vol. 4 . Academic Press, New York, pp. 407–515 Physiology, Part I

[ref8] Beck MW, Brumbaugh RD, Airoldi L, Carranza A, Coen LD, Crawford C, Defeo O, Edgar GJ, Hancock B, Kay MC et al. (2011) Oyster reefs at risk and recommendations for conservation, restoration, and management. Bioscience 61: 107–116.

[ref9] Berger VJ, Kharazova AD (1997) Mechanisms of salinity adaptations in marine molluscs. In AD Naumov, H Hummel, AA Sukhotin, JS Ryland, eds, Interactions and Adaptation Strategies of Marine Organisms (Developments in Hydrobiology), Vol 121. Springer, Dordrecht

[ref10] Bernatchez S, Xuereb A, Laporte M, Benestan L, Steeves R, Laflamme M, Bernatchez L, Mallet MA (2019) Seascape genomics of eastern oyster (*Crassostrea virginica*) along the Atlantic coast of Canada. Evol Appl 12: 587–609. 30828376 10.1111/eva.12741 PMC6383708

[ref11] Beseres Pollack J, Kim H-C, Morgan EK, Montagna PA (2011) Role of flood disturbance in natural oyster (*Crassostrea virginica*) population maintenance in an estuary in South Texas, USA. Estuaries Coast 34: 187–197.

[ref12] Biasutti M, Sobel AH, Camargo SJ, Creyts TT (2012) Projected changes in the physical climate of the Gulf Coast and Caribbean. Clim Change 112: 819–845.

[ref13] Bible JM, Sanford E (2016) Local adaptation in an estuarine foundation species: implications for restoration. Biol Conserv 193: 95–102.

[ref14] Breuer JP (1962) An ecological survey of the lower Laguna Madre of Texas, 1953–1959. Pub Inst Mar Sci Univ Texas 8: 3–183.

[ref15] Brown BL, Butt AJ, Shelton SW, Paynter KT (1998) Growth and mortality of North Carolina-Heritage oysters, *Crassostrea virginica*, in North Carolina and in Chesapeake Bay. J Appl Aquac 8: 25–39.

[ref16] Burford MO, Scarpa J, Cook BJ, Hare MP (2014) Local adaptation of a marine invertebrate with a high dispersal potential: evidence from a reciprocal transplant experiment of the eastern oyster *Crassostrea virginica*. Mar Ecol Prog Ser 505: 161–175.

[ref17] Burnham KP, Anderson DR (1998) Practical use of the information-theoretic approach. In Model Selection and Inference. Springer, New York, NY, pp. 75–117

[ref18] Buroker NE (1983) Population genetics of the American oyster *Crassostrea virginica* along the Atlantic coast and the Gulf of Mexico. Mar Biol 75: 99–112.

[ref19a] Bushek D, Ford SE, Allen Jr SK (1994) Evaluation of methods using Ray's fluid thioglycollate medium for diagnosis of Perkinsus marinus infection in the eastern oyster, Crassostrea virginica. Ann Rev Fish 4: 201–217.

[ref19] Bushek D, Ford SE, Burt I (2012) Long-term patterns of an estuarine pathogen along a salinity gradient. J Mar Res 70: 225–251.

[ref20] Butler PA (1949) Gametogenesis in the oyster under conditions of depressed salinity. Biol Bull 96: 263–269. 18153114

[ref21] Butler PA (1952) Effect of Floodwaters on Oysters in Mississippi Sound in 1950, Vol. 31. U.S. Government Printing Office, Washington, DC, 20 pp, U.S. Fish and Wildlife Service and U.S. Department of the Interior Research Report 31

[ref22] Carter L, Terando A, Dow K, Hiers K, Kunkel KE, Lascurain A, Marcy D, Osland M, Schramm P (2018) Southeast. In DR Reidmiller, CW Avery, DR Easterling, KE Kunkel, KLM Lewis, TK Maycock, BC Stewart, eds, Impacts, Risks, and Adaptation in the United States: Fourth National Climate Assessment, Volume II. U.S. Global Change Research Program, Washington, DC, USA, pp. 743–808

[ref23] Casas S, Walton W, Chapline G, Rikard S, Supan J, La Peyre J (2017) Performance of oysters selected for dermo resistance compared to wild oysters in northern Gulf of Mexico estuaries. Aquac Environ Interact 9: 169–180.

[ref24] Casas SM, Lavaud R, La Peyre MK, Comeau LA, Filgueira R, La Peyre JF (2018) Quantifying salinity and season effects on eastern oyster clearance and oxygen consumption rates. Mar Biol 165: 90.

[ref25] Chu FLE, La Peyre JF, Burreson CS (1993) *Perkinsus marinus* infection and potential defense-related activities in eastern oysters, *Crassostrea virginica*: salinity effects. J Invertebr Pathol 62: 226–232.

[ref26] Coen LD, Brumbaugh RD, Bushek D, Grizzle R, Luckenbach MW, Posey MH, Powers SP, Tolley SG (2007) Ecosystem services related to oyster restoration. Mar Ecol Prog Ser 341: 303–307.

[ref27] Conover DO, Clarke LM, Munch SB, Wagner GN (2006) Spatial and temporal scales of adaptive divergence in marine fishes and the implications for conservation. J Fish Biol 69: 21–47.

[ref28] Craig A, Powell EN, Fay RR, Brooks JM (1989) Distribution of *Perkinsus marinus* in gulf coast oyster populations. Estuaries 12: 82–91.

[ref29] Dittman DE, Ford SE, Haskin HH (1998) Growth patterns in oysters, *Crassostrea virginica*, from different estuaries. Mar Biol 132: 461–469.

[ref30] Donelan SC, Breitburg D, Ogburn MB (2021) Context-dependent carryover effects of hypoxia and warming in a coastal ecosystem engineer. Ecol Appl 31: 1–15. 10.1002/eap.2315. PMC8243920 33636022

[ref31] Eierman LE, Hare MP (2013) Survival of oyster larvae in different salinities depends on source population within an estuary. J Exp Mar Biol Ecol 449: 61–68.

[ref32] Eierman LE, Hare MP (2014) Transcriptomic analysis of candidate osmoregulatory genes in the eastern oyster *Crassostrea virginica*. BMC Genomics 15: 1–15. 24950855 10.1186/1471-2164-15-503 PMC4101419

[ref33] Eierman LE, Hare MP (2016) Reef-specific patterns of gene expression plasticity in eastern oysters (*Crassostrea virginica*). J Hered 107: 90–100. 26245921 10.1093/jhered/esv057

[ref34] Fisher WS, Oliver LM (1996) A whole-oyster procedure for diagnosis of *Perkinsus marinus* disease using Ray's fluid thioglycollate culture medium. J Shellfish Res 15: 109–118.

[ref35] Fuhrmann M, Delisle L, Petton B, Corporeau C, Pernet F (2018) Metabolism of the Pacific oyster, *Crassostrea gigas*, is influenced by salinity and modulates survival to the Ostreid herpesvirus OsHV-1. Biol Open 7: bio028134. 10.1242/bio.028134. PMC5861354 29463513

[ref36] Griffiths JS, Johnson KM, Sirovy KA, Yeats MS, Pan FT, La Peyre JF, Kelly MW (2021) Transgenerational plasticity and the capacity to adapt to low salinity in the eastern oyster, *Crassostrea virginica*. Proc Biol Sci 288: 20203118. 34004136 10.1098/rspb.2020.3118 PMC8131124

[ref37] Groue KJ, Lester LJ (1982) A morphological and genetic analysis of geographic variation among oysters in the Gulf of Mexico. Veliger 24: 331–335.

[ref38] Hand SC, Stickle WB (1977) Effects of tidal fluctuations of salinity on pericardial fluid composition of the American oyster *Crassostrea virginica*. Mar Biol 42: 259–271.

[ref39] Hayes PF, Menzel RW (1981) The reproductive cycle of early setting *Crassostrea virginica* (Gmelin) in the northern Gulf of Mexico, and its implications for population recruitment. Biol Bull 160: 80–88.

[ref40] Henry RP, Mangum CP, Webb KL (1980) Salt and water balance in the oligohaline clam, Rangia cuneata II. Accumulation of intracellular free amino acids during high salinity adaptation. J Exp Zool 211: 11–24.

[ref41] Heavers BW, Hammen CS (1985) Fate of endogenous free amino acids in osmotic adjustment of *Crassostrea virginica* (Gmelin). Comp Biochem Physiol A 82: 571–576.

[ref42] Hoover CA, Gaffney PM (2005) Geographic variation in nuclear genes of the eastern oyster, *Crassostrea virginica* Gmelin. J Shellfish Res 24: 103–112.

[ref43] Huvet A, Normand J, Fleury E, Quillien V, Fabioux C, Boudry P (2010) Reproductive effort of Pacific oysters: a trait associated with susceptibility to summer mortality. Aquaculture 304: 95–99.

[ref44] Johnson KM, Kelly MW (2020) Population epigenetic divergence exceeds genetic divergence in the eastern oyster *Crassostrea virginica* in the northern Gulf of Mexico. Evol Appl 13: 945–959. 32431745 10.1111/eva.12912 PMC7232765

[ref45] Johnson KM, Sirovy KA, Casas SM, La Peyre JF, Kelly MW (2020) Characterizing the epigenetic and transcriptomic responses to *Perkinsus marinus* infection in the eastern oyster *Crassostrea virginica*. Front Mar Sci 7: 598. 10.3389/fmars.2020.00598.

[ref46] Kawecki TJ, Ebert D (2004) Conceptual issues in local adaptation. Ecol Lett 7: 1225–1241.

[ref47] King TL, Ward R, Zimmerman EG (1994) Population structure of eastern oysters (*Crassostrea virginica*) inhabiting the Laguna Madre, Texas, and adjacent bay systems. Can J Fish Aquat Sci 51: 215–222.

[ref48] Kloesel K, Bartush B, Banner J, Brown D, Lemory J, Lin X, McManus G, Mullens E, Nielsen-Gammon J, Shafer M et al. (2018) Southern Great Plains. In DR Reidmiller, CW Avery, DR Easterling, KE Kunkel, KLM Lewis, TK Maycock, BC Stewart, eds, Impacts, Risks, and Adaptation in the United States: Fourth National Climate Assessment, Volume II. U.S. Global Change Research Program, Washington, DC, USA, pp. 987–1035

[ref49] Kraeuter JN, Ford S, Cummings M (2007) Oyster growth analysis: a comparison of methods. J Shellfish Res 26: 479–491.

[ref50] La Peyre M, Casas S, La Peyre J (2006) Salinity effects on viability, metabolic activity and proliferation of three *Perkinsus* species. Dis Aquat Organ 71: 59–74. 16922001 10.3354/dao071059

[ref51] La Peyre MK, Nickens AD, Volety AK, Tolley GS, La Peyre JF (2003) Environmental significance of freshets in reducing *Perkinus marinus* infection in eastern oysters *Crassostrea virginica*: potential management applications. Mar Ecol Prog Ser 248: 165–176.

[ref52] La Peyre MK, Gossman B, La Peyre JF (2009) Defining optimal freshwater flow for oyster production: effects of freshet rate and magnitude of change and duration on eastern oysters and *Perkinsus marinus* infection. Estuaries Coast 32: 522–534.

[ref53] La Peyre MK, Eberline BS, Soniat TM, La Peyre JF (2013) Differences in extreme low salinity timing and duration differentially affect eastern oyster (*Crassostrea virginica*) size class growth and mortality in Breton Sound, LA. Estuar Coast Shelf Sci 135: 146–157.

[ref54] La Peyre MK, Humphries AT, Casas SM, La Peyre JF (2014) Temporal variation in development of ecosystem services from oyster reef restoration. Ecol Eng 63: 34–44.

[ref55] La Peyre MK, Aguilar Marshall D, Miller LS, Humphries AT (2019a) Oyster reefs in northern Gulf of Mexico estuaries harbor diverse fish and decapod crustacean assemblages: a meta-synthesis. Front Mar Sci 6: 666.

[ref56] La Peyre JF, Casas SM, Richards M, Xu W, Xue Q (2019b) Testing plasma subtilisin inhibitory activity as a selective marker for dermo resistance in eastern oysters. Dis Aquat Organ 133: 127–139. 31019137 10.3354/dao03344

[ref57] Lavaud R, La Peyre MK, Casas SM, Bacher C, La Peyre JF (2017) Integrating the effects of salinity on the physiology of the eastern oyster, *Crassostrea virginica*, in the northern Gulf of Mexico through a Dynamic Energy Budget model. Ecol Model 363: 221–233.

[ref58] Lavaud R, La Peyre MK, Justic D, La Peyre JF (2021) Dynamic Energy Budget modelling to predict eastern oyster growth, reproduction, and mortality under river management and climate change scenarios. Estuar Coast Shelf Sci 251: 107–188.

[ref59] Leonhardt JM, Casas S, Supan JE, La Peyre JF (2017) Stock assessment for eastern oyster seed production and field grow-out in Louisiana. Aquaculture 466: 9–19.

[ref60] Li L, Li AO, Song K, Meng J, Guo X, Li S, Li C, de Wit P, Que H, Wu F et al. (2018) Divergence and plasticity shape adaptive potential of the Pacific oyster. Nat Ecol Evol 2: 1751–1760. 30250157 10.1038/s41559-018-0668-2

[ref61] Livingston RJ, Lewis FG, Woodsum GC, Niu XF, Galperin B, Huang W, Christensen JD, Monaco ME, Battista TA, Klein CJ et al. (2000) Modelling oyster population response to variation in freshwater input. Estuar Coast Shelf Sci 50: 655–672.

[ref62] Loosanoff VL (1953) Behavior of oysters in water of low salinities. Proc. Natl Shellfish Ass 43: 135–151.

[ref63] Lord J, Whitlatch R (2014) Latitudinal patterns of shell thickness and metabolism in the eastern oyster *Crassostrea virginica* along the east coast of North America. Mar Biol 161: 1487–1497.

[ref64] LDWF (2018) Oyster stock assessment report. Oyster data report Series No. 24. Louisiana Department of Wildlife and Fisheries P.O. Box 98000, Baton Rouge, LA 70998.

[ref65] Lowe MR, Sehlinger T, Soniat TM, La Peyre MK (2017) Interactive effects of water temperature and salinity on growth and mortality of eastern oysters, *Crassostrea virginica*: a meta-analysis using 40 years of monitoring data. J Shellfish Res 36: 683–697.

[ref66] Mackin JG (1962) Oyster disease caused by *Dermocystidium marinum* and other microorganisms in Louisiana. Pub Inst Mar Sci Univ Texas 7: 132–229.

[ref67] Martinez-Andrade F (2018) Trends in Relative Abundance and Size of Selected Finfishes and Shellfishes along the Texas Coast: November 1975–December 2016. Texas Parks and Wildlife Department, Management Data Series, no. 293. 10.13140/RG.2.2.14487.06565.

[ref68] Maynard A, Bible JM, Pespeni MH, Sanford E, Evans TG (2018) Transcriptomic responses to extreme low salinity among locally adapted populations of Olympia oyster (*Ostrea lurida*). Mol Ecol 27: 4225–4240. 30193406 10.1111/mec.14863

[ref69] McCarty AJ, McFarland K, Small J, Allen SK Jr, Plough LV (2020) Heritability of acute low salinity survival in the Eastern oyster (*Crassostrea virginica*). Aquaculture 529: 735649.

[ref70] McKay JK, Christian CE, Harrison S, Rice KJ (2005) “How local is local?”—A review of practical and conceptual issues in the genetics of restoration. Restor Ecol 13: 432–440.

[ref71] Meng J, Zhu Q, Zhang L, Li C, Li L, She Z, Huang B, Zhang G (2013) Genome and transcriptome analyses provide insight into the euryhaline adaptation mechanism of *Crassostrea gigas*. PLoS One 8: e58563. 10.1371/journal.pone.0058563. 23554902 PMC3595286

[ref72] Méthé D, Comeau LA, Stryhn H, Landry T, Davidson J (2015) Stress response of *Crassostrea virginica* (Gmelin, 1791) oysters following a reciprocal transfer between upriver and downriver sites. Aquacult Res 46: 2841–2850.

[ref73] Montagna PA, Palmer TA, Beseres Pollack J (2013) Hydrological Changes and Estuarine Dynamics. Springer, New York.

[ref74] Munroe D, Tabatabai A, Burt I, Bushek D, Powell EN, Wilkin J (2013) Oyster mortality in Delaware Bay: impacts and recovery from hurricane Irene and tropical storm Lee. Estuar Coast Shelf Sci 135: 209–219.

[ref75] NOAA (2020) Commercial Fisheries Statistics, Annual Landings. https://www.st.nmfs.noaa.gov/commercial-fisheries/commercial-landings/annual-landings/index. (date last accessed, 1 April 2020).

[ref76] Nevins JA, Beseres Pollack J, Stunz GW (2014) Characterizing nekton use of the largest unfished oyster reef in the United States compared with adjacent estuarine habitats. J Shellfish Res 33: 227–238.

[ref77] Orlando SP Jr, Rozas LP, Ward GH, Klein CJ (1993) Characteristics of Gulf of Mexico Estuaries. National and Atmospheric Administration, Office of Ocean Resources Conservation and Assessment, Silver Spring

[ref78] Parker LM, Ross PM, O’Connor WA (2011) Populations of the Sydney rock oyster, *Saccostrea glomerate*, vary in response to ocean acidification. Mar Biol 158: 689–697.

[ref79] Petes LE, Brown AJ, Knight CR (2012) Impacts of upstream drought and water withdrawals on the health and survival of downstream estuarine oyster populations. Ecol Evol 2: 1712–1724. 22957175 10.1002/ece3.291 PMC3434945

[ref80] Pierce SK (1982) Invertebrate cell volume control mechanisms: a coordinated use of intracellular amino acids and inorganic ions as osmotic solute. Biol Bull 163: 405–419.

[ref81] Pierce SK, Rowland-Faux LM, O'Brien SM (1992) Different salinity tolerance mechanisms in Atlantic and Chesapeake Bay conspecific oysters: glycine betaine and amino acid pool variations. Mar Biol 113: 107–115.

[ref82] Pierce SK, Rowland-Faux LM, Crombie BN (1995) The mechanism of glycine betaine regulation in response to hyperosmotic stress in oyster mitochondria: a comparative study of Atlantic and Chesapeake Bay oysters. J Exp Zool 271: 161–170.

[ref83] Powell EJ, Keim BD (2015) Trends in daily temperature and precipitation extremes for the southeastern United States: 1948-2012. J Climate 28. 10.1175/JCLI-D-14-00410.1.

[ref84] Prein AF, Rasmussen RM, Ikeda K, Liu C, Clark MP, Holland GJ (2017) The future intensification of hourly precipitation extremes. Nat Clim Change 7: 48–52.

[ref85] R Foundation for Statistical Computing (2019) R: A Language and Environment for Statistical Computing. R Foundation for Statistical Computing, Vienna Austria

[ref86] Ragone Calvo LM, Dungan CF, Roberson BS, Burreson EM (2003) Systematic evaluation of factors controlling *Perkinsus marinus* transmission dynamics in lower Chesapeake Bay. Dis Aquat Organ 56: 75–86. 14524504 10.3354/dao056075

[ref87] Rainer JS, Mann R (1992) A comparison of methods for calculating condition index in eastern oysters *Crassostrea virginica* (Gmelin, 1791). J Shellfish Res 11: 55–58.

[ref88] Reeb CA, Avise JC (1990) A genetic discontinuity in a continuously distributed species: mitochondrial DNA in the American oyster, *Crassostrea virginica*. Genetics 124: 397–406. 1968412 10.1093/genetics/124.2.397 PMC1203931

[ref89] Riekenberg J, Bargu S, Twilley R (2015) Phytoplankton community shifts and harmful algae presence in a diversion influenced estuary. Estuaries Coast 38: 2213–2226.

[ref90] Rybovich M, La Peyre MK, Hall SG, La Peyre JF (2016) Increased temperatures combined with lowered salinities differentially impact oyster size class growth and mortality. J Shellfish Res 35: 101–113.

[ref91] Sanford E, Kelly MW (2011) Local adaptation in marine invertebrates. Ann Rev Mar Sci 3: 509–535. 10.1146/annurev-marine-120709-142756 21329215

[ref92] Scharping RJ, Plough LV, Meritt DW, North EW (2019) Low-salinity tolerance of early-stage oyster larvae from a mesohaline estuary. Mar Ecol Prog Ser 613: 97–106.

[ref93] Scherer AE, Garcia MM, Smee DL (2017) Predatory blue crabs induce stronger nonconsumptive effects in eastern oysters *Crassostrea virginica* than scavenging blue crabs. PeerJ 5: e3042. 10.7717/peerj.3042. 28265512 PMC5333538

[ref94] Sehlinger T, Lowe MR, La Peyre MK, Soniat TM (2019) Differential effects of temperature and salinity on growth and mortality of oysters (*Crassostrea virginica*) in Barataria Bay and Breton Sound, Louisiana. J Shellfish Res 38: 317–326.

[ref95] Shumway SE (1977) Effect of salinity fluctuation on the osmotic pressure and Na^+^, Ca^+^ and Mg^2+^ ion concentrations in the hemolymph of bivalve molluscs. Mar Biol 41: 153–177.

[ref96] Shumway SE (1996) Natural environmental factors. In VS Kennedy, RIE Newell, AF Ebele, eds, The Eastern Oyster, Crassostrea virginica. Maryland Sea Grand, College Park, Maryland, U.S.A.

[ref97] Shumway SE, Koehn RK (1982) Oxygen consumption in the American oyster *Crassostrea virginica*. Mar Ecol Prog Ser 9: 59–68.

[ref98] Sokolova IM, Frederich M, Bagwe R, Lannig G, Sukhotin AA (2012) Energy homeostasis as an integrative tool for assessing limits of environmental stress tolerance in aquatic invertebrates. Mar Environ Res 79: 1–15. 22622075 10.1016/j.marenvres.2012.04.003

[ref99] Soniat TM, Finelli CM, Ruiz JT (2004) Vertical structure and predator refuge mediate oyster reef development and community dynamics. J Exp Mar Biol Ecol 310: 163–182.

[ref100] Soniat TM, Conzelmann CP, Byrd JD, Roszell DP, Bridevaux JL, Suir KJ, Colley SB (2013) Predicting the effects of proposed Mississippi River diversions on oyster habitat quality; application of an oyster habitat suitability index model. J Shellfish Res 32: 629–638.

[ref101] Spencer LH, Venkataraman YR, Crim R, Ryan S, Horwith MJ, Roberts SB (2020) Carryover effects of temperature and pCO _2_ across multiple Olympia oyster populations. Ecol Appl 30: e02060. 10.1002/eap.2060. 31863716

[ref102] Southworth M, Long MC, Mann R (2017) Oyster (*Crassostrea virginica* [Gmelin, 1791]) mortality at prolonged exposures to high temperature and low salinity. J Shellfish Res 36: 335–340.

[ref103] Sukhotin AA, Lajus DL, Lesin PA (2003) Influence of age and size on pumping activity and stress resistance in the marine bivalve *Mytilus edulis* L. J of Exp Mar Biol Ecol 284: 129–144.

[ref104] Supan JE, Wilson CA (2001) Analyses of gonadal cycling by oyster broodstock, *Crassostrea virginica* (Gmelin), in Louisiana. J Shellfish Res 20: 215–220.

[ref105] Turley B, Reece K, Shen J, Lee JH, Guo X, McDowell J (2019) Multiple drivers of interannual oyster settlement and recruitment in the lower Chesapeake Bay. Conserv Genet 20: 1057–1071.

[ref106] Varney RL, Galindo-Sánchez CE, Cruz P, Gaffney PM (2009) Population genetics of the eastern oyster *Crassostrea virginica* (Gmelin, 1791) in the Gulf of Mexico. J Shellfish Res 28: 855–864.

[ref107] Volety AK, Haynes L, Goodman P, Gorman P (2014) Ecological condition and value of oyster reefs of the Southwest Florida shelf ecosystem. Ecol Indic 44: 108–119.

[ref108] Wadsworth P, Casas S, La Peyre J, Walton W (2019) Elevated mortalities of triploid eastern oysters cultured off-bottom in northern Gulf of Mexico. Aquaculture 505: 363–373.

[ref109] Wallace RK, Waters P, Rikard FS (2008) Oyster hatchery techniques. Southern Regional Aquaculture Center Publication No 4302.

[ref110] Wang H, Huang W, Harwell MA, Edmiston L, Johnson E, Hsieh P, Milla K, Christensen J, Stewart J, Liu X (2008) Modeling oyster growth rate by coupling oyster population and hydrodynamic models for Apalachicola Bay, Florida, USA. Ecol Model 211: 77–89.

[ref111] Wang, Chen Q, La Peyre MK, Hu K, La Peyre JF (2017) Predicting the impacts of Mississippi River diversions and sea-level rise on spatial patterns of eastern oyster growth rate and production. Ecol Model 352: 40–53.

[ref112] Wetz MS, Yoskowitz DW (2013) An ‘extreme’ future for estuaries? Effects of extreme climatic events on estuarine water quality and ecology. Mar Pollut Bull 69: 7–18. 23474351 10.1016/j.marpolbul.2013.01.020

[ref113] Wheeler MW, Park RM, Bailer AJ (2006) Comparing median lethal concentration values using confidence interval overlap or ratio tests. Environ Toxicol Chem 25: 1441–1444. 16704080 10.1897/05-320r.1

[ref114] Wilber DH (1992) Associations between freshwater inflows and oyster productivity in Apalachicola Bay, Florida. Estuar Coast Shelf Sci 35: 179–190.

[ref115] Wilbur AE, Gaffney PM (1997) A genetic basis for geographic variation in shell morphology in the bay scallop, *Argopecten irradians*. Mar Biol 128: 97–105.

[ref116] Widdows J (1978) Physiological indexes of stress in *Mytilus-edulis*. J Mar Biolog 58: 125–142.

[ref117] Yuan W, Walters LJ, Schneider KR, Hoffman EA (2010) Exploring the survival threshold: a study of salinity tolerance of the nonnative mussel *Mytella charruana*. J Shellfish Res 29: 415–422.

[ref118] Zhang G, Fang X, Guo X, Li LI, Luo R, Xu F, Yang P, Zhang L, Wang X, Qi H et al. (2012) The oyster genome reveals stress adaptation and complexity of shell formation. Nature 490: 49–54. 22992520 10.1038/nature11413

