# Supplementary material for: Divergence in salinity tolerance of northern Gulf of Mexico eastern oysters under field and laboratory exposure
Source: Conserv Physiol. 2021 Aug 23;9(1):coab065. doi: 10.1093/conphys/coab065 (PMC8384081; doi:10.1093/conphys/coab065)
Supplement: Supplemental_Material_coab065 [file supplemental_material_coab065.docx]

# Supplemental Material

Supplemental Table 1. Initial mean (± SD) shell heights of the progenies of the four oyster broodstocks (PC=Packery Channel, AB=Aransas Bay, CL=Calcasieu Lake, VB=Vermilion Bay) deployed at the Alabama Grand Bay and Mobile Bay sites in December 2018. Stocks at either site with different letters are statistically different (<0.05).

| Site | Stock | Initial shell height (mm) | | | |
| --- | --- | --- | --- | --- | --- |
| Grand Bay | PC | 32.6 | ± | 4.7 | A |
|  | AB | 29.0 | ± | 4.0 | B |
|  | CL | 27.5 | ± | 3.3 | D |
|  | VB | 29.8 | ± | 3.5 | B |
| Mobile Bay | PC | 33.6 | ± | 4.5 | A |
|  | AB | 29.4 | ± | 3.9 | BC |
|  | CL | 26.3 | ± | 3.0 | CD |
|  | VB | 29.3 | ± | 3.6 | B |

Supplemental Table 2. Sampling schedule for the field study. The progenies of the four oysters broodstocks were deployed from December 2018 to October 2019 at Alabama Mobile Bay and Grand Bay. Analyses on salinity and temperature, mortality, and shell height (i.e., growth) are presented within the main text in intervals (Dec-Apr, Apr-Jul, Jul-Oct).

| Date | Field salinity, temperature | Mortality (%) | Shell height (mm) | Condition index | *P. marinus* infection intensity (parasites g^-1^ wet tissue) |
| --- | --- | --- | --- | --- | --- |
| Dec-18 | x | x | x |  |  |
| Jan-19 | x | x | x |  |  |
| Feb-19 | x | x | x |  |  |
| Mar-19 | x | x | x |  |  |
| Apr-19 | x | x | x | x | x |
| May-19 | x | x | x |  |  |
| Jun-19 | x | x | x |  |  |
| Jul-19 | x | x | x | x | x |
| Aug-19 | x | x | x |  |  |
| Sep-19 | x | x | x |  |  |
| Oct-19 | x | x | x | x | x |

Supplemental Table 3. Interval growth rate (mm month^-1^ ) of the progenies of the four oyster broodstocks (PC=Packery Channel, AB=Aransas Bay, CL=Calcasieu Lake, VB=Vermilion Bay) at the Alabama Grand Bay and Mobile Bay sites with daily mean (± SD) salinity and temperature during each interval.

| Site | Interval | Stock | Growth rate (mm month^-1^) | | | Salinity | Temperature (°C) |
| --- | --- | --- | --- | --- | --- | --- | --- |
| Grand Bay | Dec-Apr | PC | 2.2 | ± | 0.4 | 15.5 ± 3.6 | 16.2 ± 2.9 |
|  |  | AB | 2.2 | ± | 0.3 |  |  |
|  |  | CL | 2.6 | ± | 0.3 |  |  |
|  |  | VB | 2.2 | ± | 0.2 |  |  |
|  | Apr-Jul | PC | 4.4 | ± | 1.4 | 12.0 ± 4.8 | 27.1 ± 3.2 |
|  |  | AB | 3.7 | ± | 0.7 |  |  |
|  |  | CL | 4.0 | ± | 0.9 |  |  |
|  |  | VB | 3.8 | ± | 0.1 |  |  |
|  | Jul-Oct | PC | 6.7 | ± | 0.6 | 18.0 ± 7.9 | 30.1 ± 1.2 |
|  |  | AB | 5.4 | ± | 0.6 |  |  |
|  |  | CL | 6.0 | ± | 0.9 |  |  |
|  |  | VB | 5.7 | ± | 0.3 |  |  |
| Mobile Bay | Dec-Apr | PC | -1.0 | ± | 0.3 | 2.2 ± 1.5 | 14.9 ± 3.6 |
|  |  | AB | 0.1 | ± | 0.2 |  |  |
|  |  | CL | 0.0 | ± | 0.2 |  |  |
|  |  | VB | -0.1 | ± | 0.3 |  |  |
|  | Apr-Jul | PC | 2.1 | ± | 0.3 | 4.5 ± 1.9 | 26.3 ± 3.6 |
|  |  | AB | 2.6 | ± | 0.7 |  |  |
|  |  | CL | 3.7 | ± | 0.2 |  |  |
|  |  | VB | 2.6 | ± | 0.8 |  |  |
|  | Jul-Oct | AB | 5.0 | ± | 0.6 | 12.8 ± 2.9 | 29.4 ± 1.5 |
|  |  | CL | 5.5 | ± | 0.3 |  |  |
|  |  | VB | 5.6 | ± | 0.7 |  |  |

Supplemental Table 4. AIC values and results of logistic regression models testing for effects of salinity, stock, and days of exposure on mortality under low and high salinity. Lowest AIC values indicate best supported models within 2 AIC units of the best model are considered to have support.

**High Salinity (22, 38, 44)**

Model AIC

**mortality ~Stock*Salinity+Day 570.95**

**mortality ~ Stock *Salinity*Day 572.26**

mortality ~Stock+Salinity*Day 581.43

mortality ~ Stock +Salinity+Day 583.91

mortality ~ Stock * Day + Salinity 585.45

mortality ~ Salinity * Day 604.12

mortality ~ Day * Stock 636.4

mortality ~ Day 654.63

mortality ~ Stock * Salinity 687.7

mortality ~ Salinity 718.71

mortality ~ Stock 745.45

Best Model: (mortality ~ Stock *Salinity+Day)

Estimate Std. Error z-value P-value

Intercept 11.808821 1.372695 8.603 < 2e-16 ***

Stock_Calcasieu_Lake 0.302086 1.737004 0.174 0.86193

Stock_Packery_Channel -3.864987 1.548530 -2.496 0.01256 *

Stock_Vermilion_Bay -0.514959 1.628117 -0.316 0.75178

Salinity -0.090132 0.033080 -2.725 0.00644 **

Day -0.046036 0.004896 -9.403 < 2e-16 ***

Stock_Calcasieu_Lake*Salinity -0.026590 0.042573 -0.625 0.53226

Stock_Packery_Channel*Salinity 0.107004 0.039924 2.680 0.00736 **

Stock_Vermilion_Bay*Salinity -0.009862 0.040124 -0.246 0.80585

**Low Salinity (2, 4, 22)**

Model AIC

**mortality ~ Stock + Salinity * Day 536.07**

**mortality ~ Day * Stock * Salinity 536.55**

**mortality ~ Stock + Salinity + Day 537.57**

mortality ~ Day * Stock + Salinity 539.24

mortality ~ Stock * Salinity + Day 542.06

mortality ~ Stock * Salinity 591.23

mortality ~ Stock * Day 597.39

mortality ~ Stock 638.06

mortality ~ Day * Salinity 670.53

mortality ~ Salinity 707.8

mortality ~ Day 720.18

Best Model: (mortality ~Stock+Salinity*Day)

Estimate Std. Err z value P-value

Intercept 8.1578586 0.4586196 17.788 < 2e-16 ***

Stock_Calcasieu_Lake 0.5293939 0.5167549 1.024 0.3056

Stock_Packery_Channel -2.3837184 0.3361849 -7.090 1.34e-12 ***

Stock_Vermilion_Bay -0.7212100 0.3877845 -1.860 0.0629

Day -0.0383718 0.0061653 -6.224 4.85e-10 ***

Salinity 0.0334695 0.0404004 0.828 0.4074

Day*Salinity 0.0016316 0.0008425 1.937 0.0528


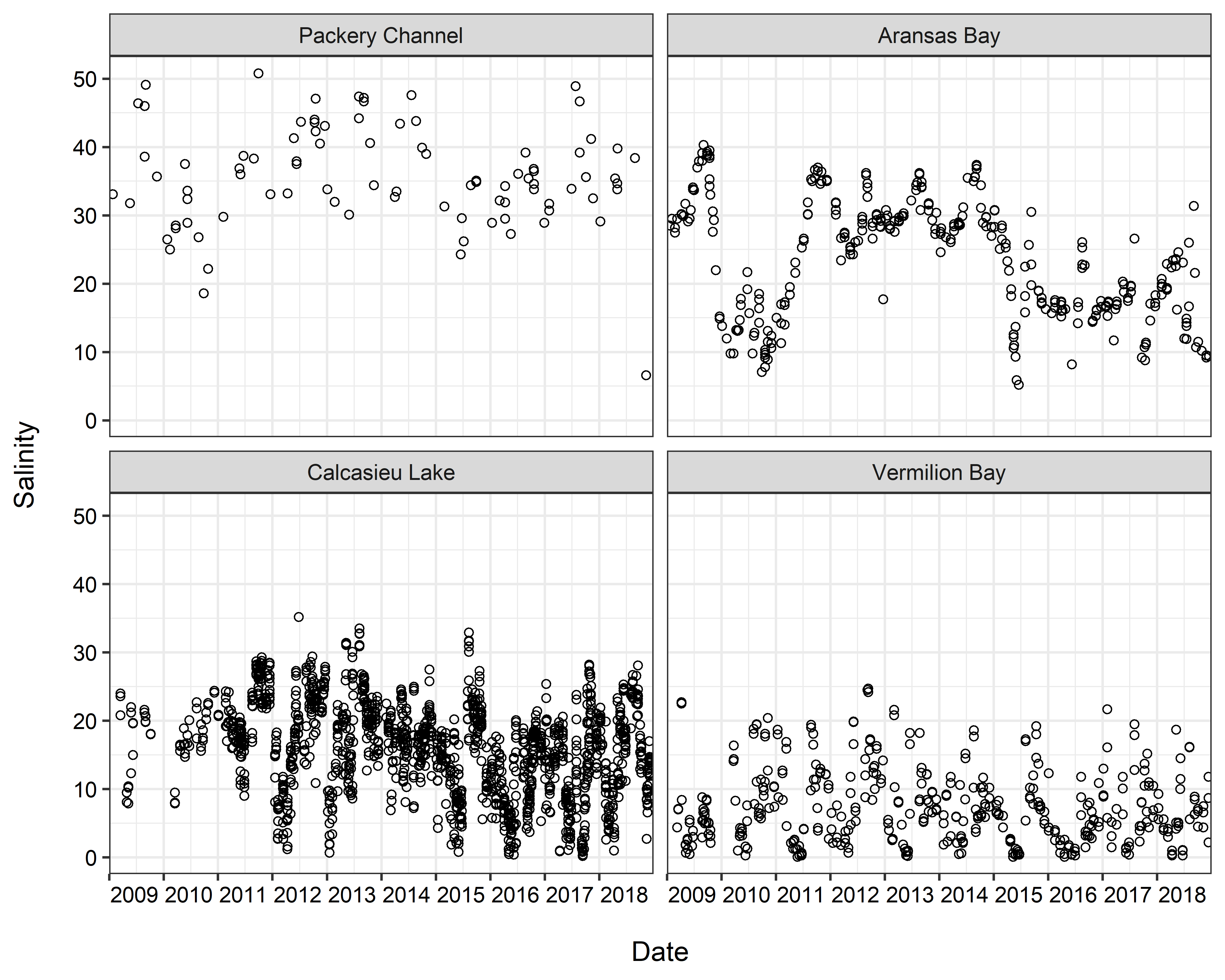


Supplemental Figure 1. Hydrological data associated with monthly (when available) oyster dredging by Louisiana Department of Wildlife and Fisheries and Texas Parks and Wildlife Department fisheries-independent monitoring program used to calculate annual salinity means for 2009-2018. Number of stations sampled are as follows: Packer Channel, Texas n=7; Aransas Bay, Texas n=6; Calcasieu Lake, Louisiana n=15; Vermilion Bay, Louisiana, n=3.


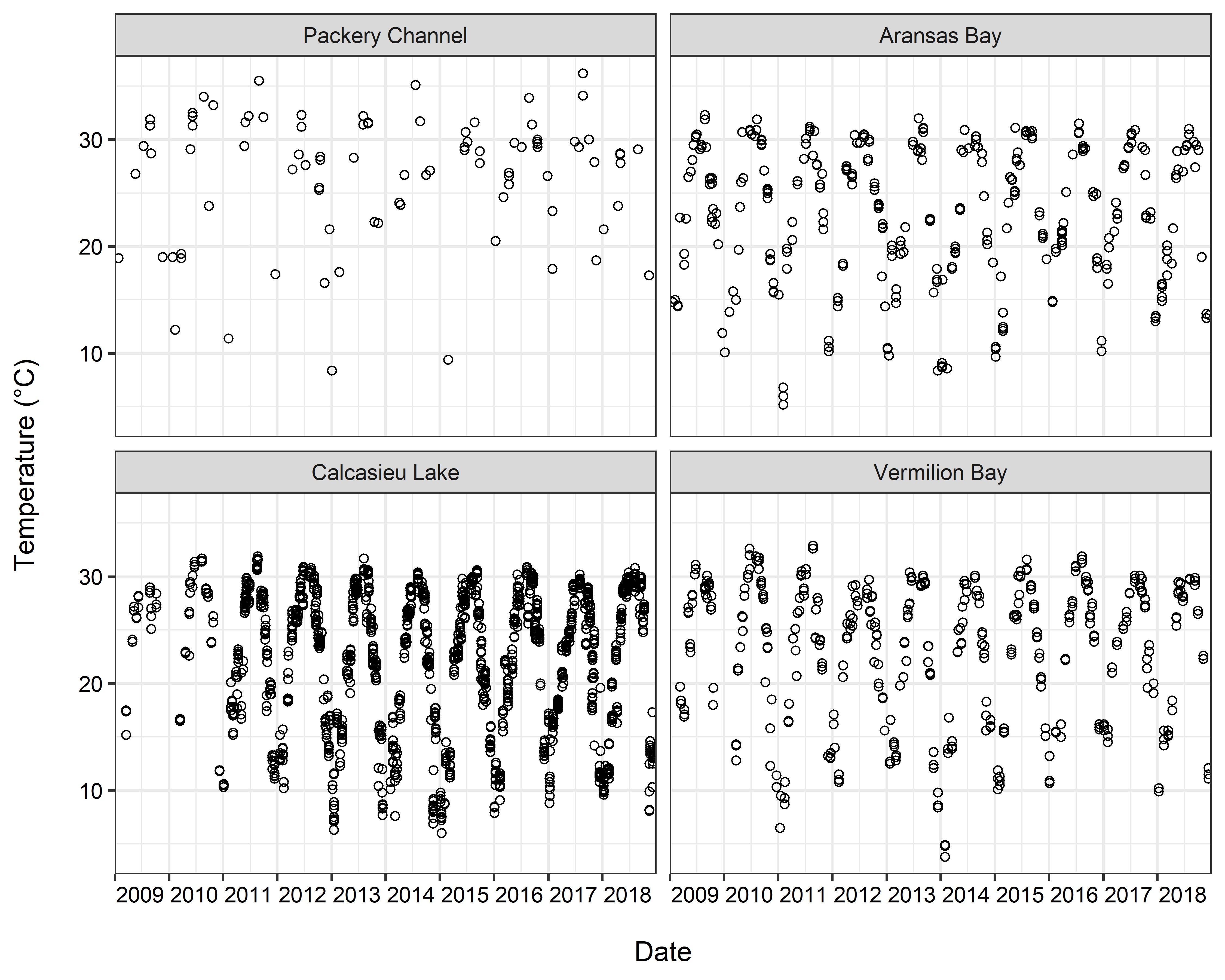


Supplemental Figure 2. Hydrological data are associated with monthly oyster dredging by Louisiana Department of Wildlife and Fisheries and Texas Parks and Wildlife Department fisheries-independent monitoring program used to calculate annual salinity means for 2009-2018. Number of stations sampled are as follows: Packer Channel, Texas n=7; Aransas Bay, Texas n=6; Calcasieu Lake, Louisiana n=15; Vermilion Bay, Louisiana n=3.


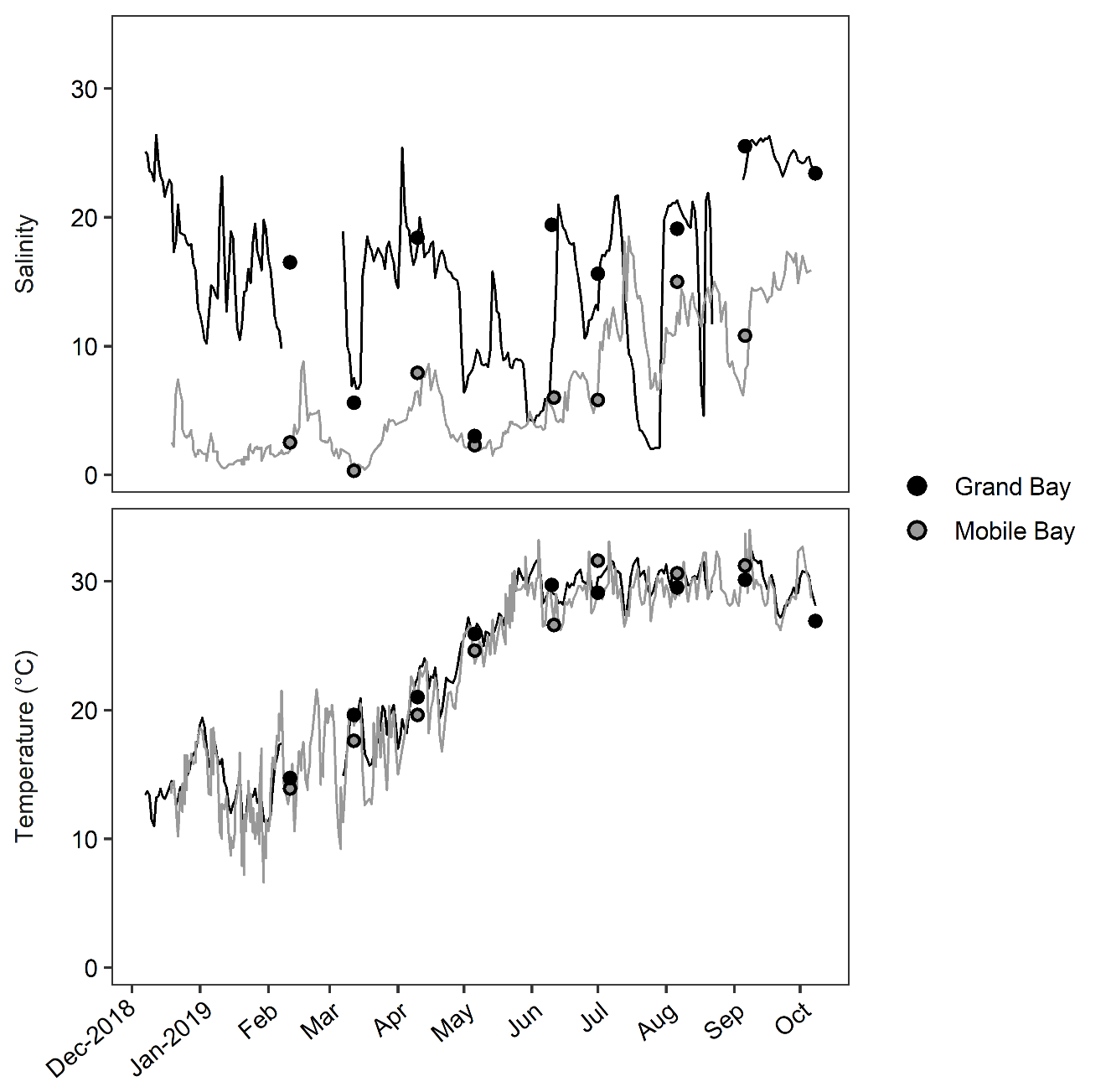


Supplemental Figure 3. Daily water temperature and salinity data from December 2018 to October 2019 at the Alabama Grand Bay (black line) and Mobile Bay (grey line) sites. Grand Bay salinity and temperature data from Point Aux Chenes (Grand Bay National Estuarine Research Reserve). Circles represent measurements at field sites on the day of sampling.


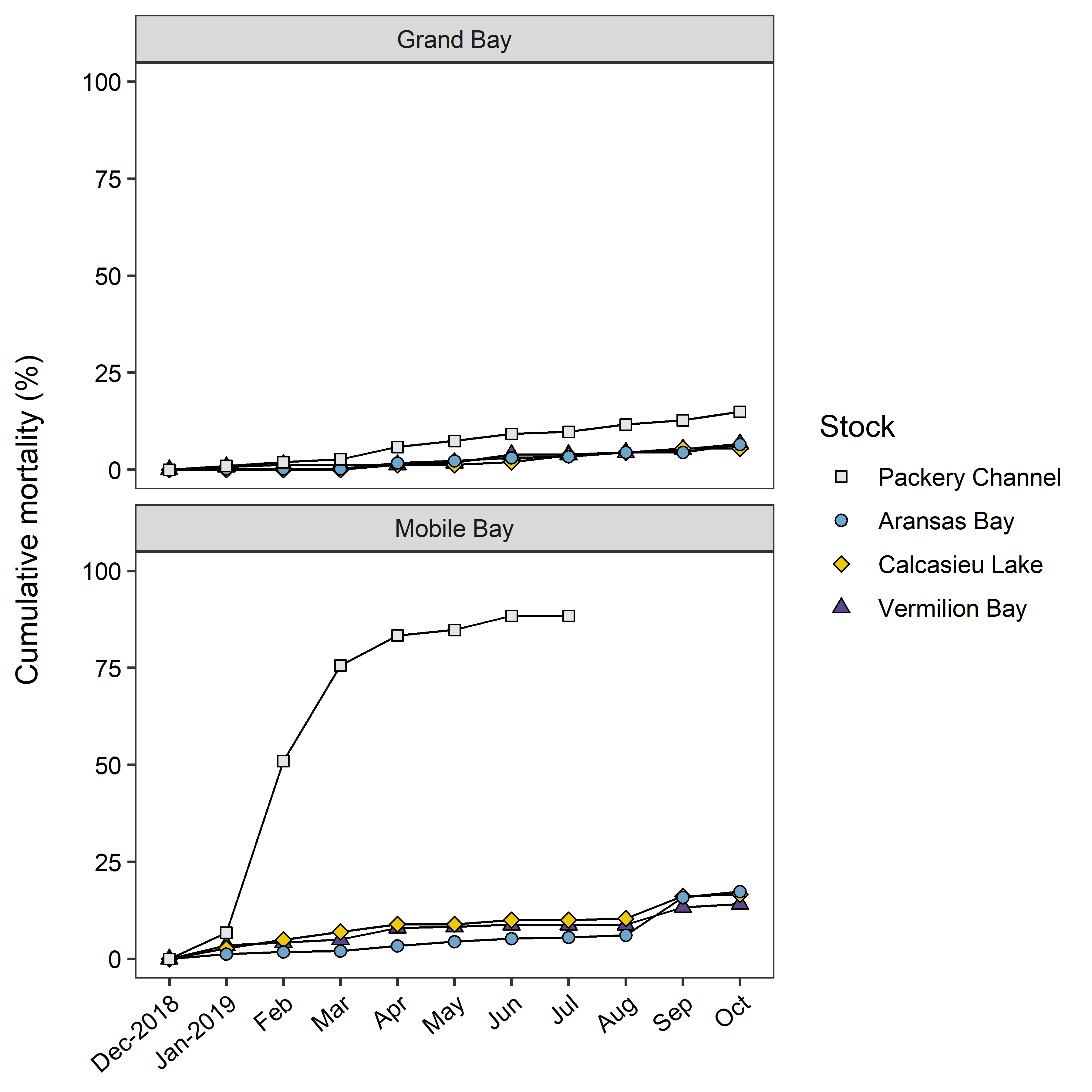


Supplemental Figure 4. Cumulative mortality of the progenies of the four oyster broodstocks at the Alabama Grand Bay and Mobile Bay sites. Replicate bags were pooled (n = 400 per stock).


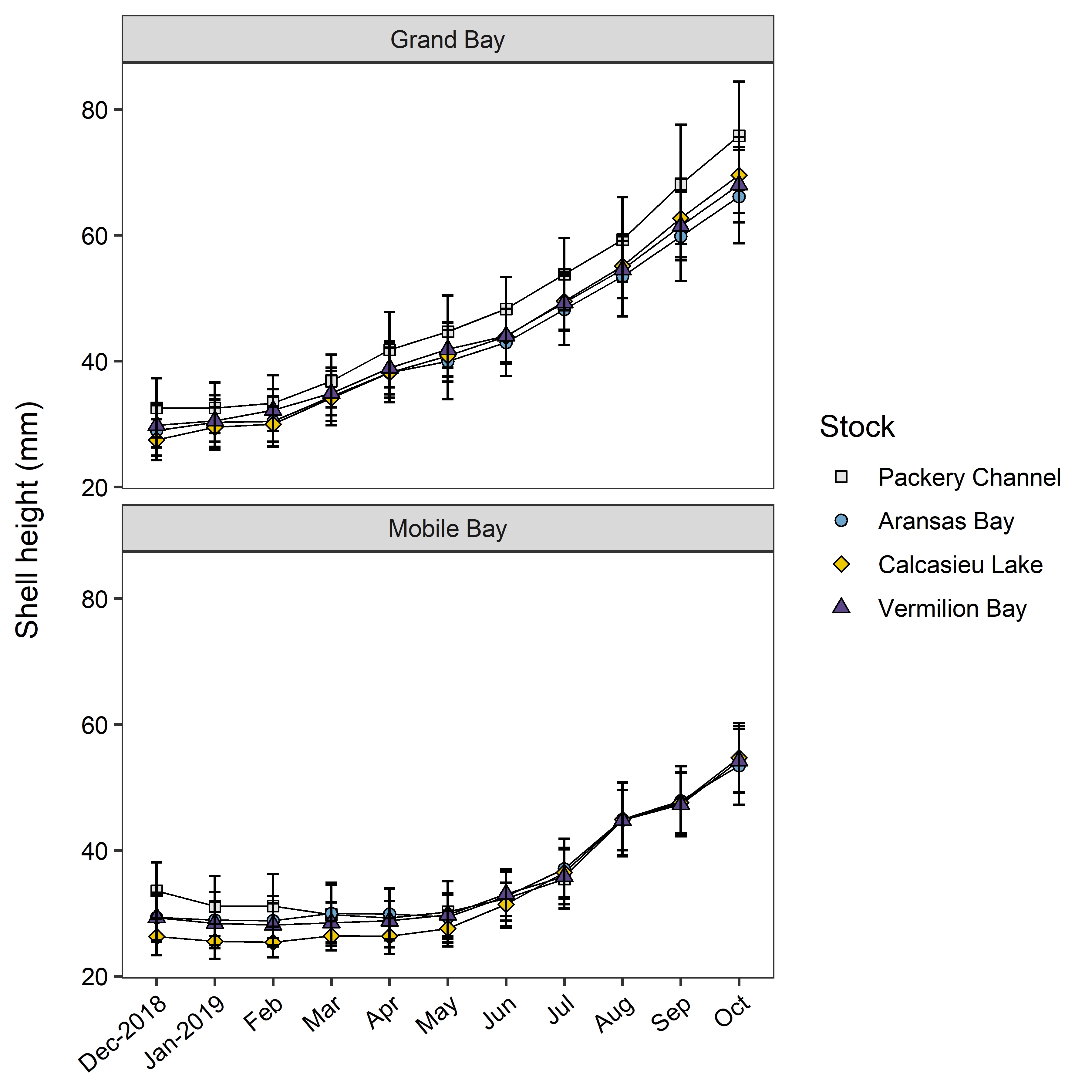


Supplemental Figure 5. Mean (± SD) shell height of the progenies of the four oyster broodstocks at the Alabama Grand Bay and Mobile Bay sites.


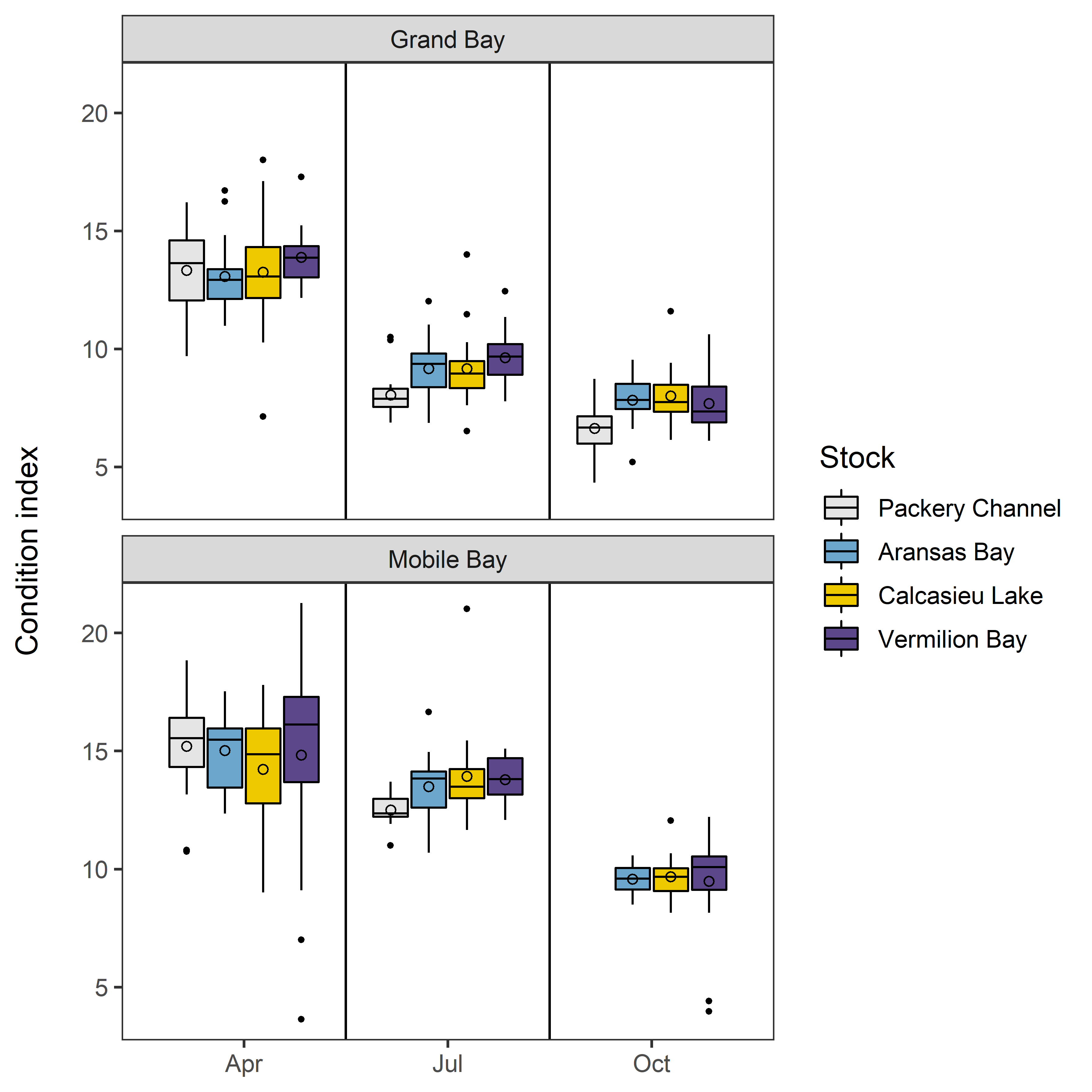


Supplemental Figure 6. Condition index of the progenies of the four oyster broodstocks at the Alabama Grand Bay and Mobile Bay sites sampled quarterly from April to October 2019. The boundaries of the box represent the 25^th^ and 75^th^ percentiles, while the line within the box is the median. Error bars indicate 1.5*IQR above and below the box boundaries, respectively. Circles represent the mean.


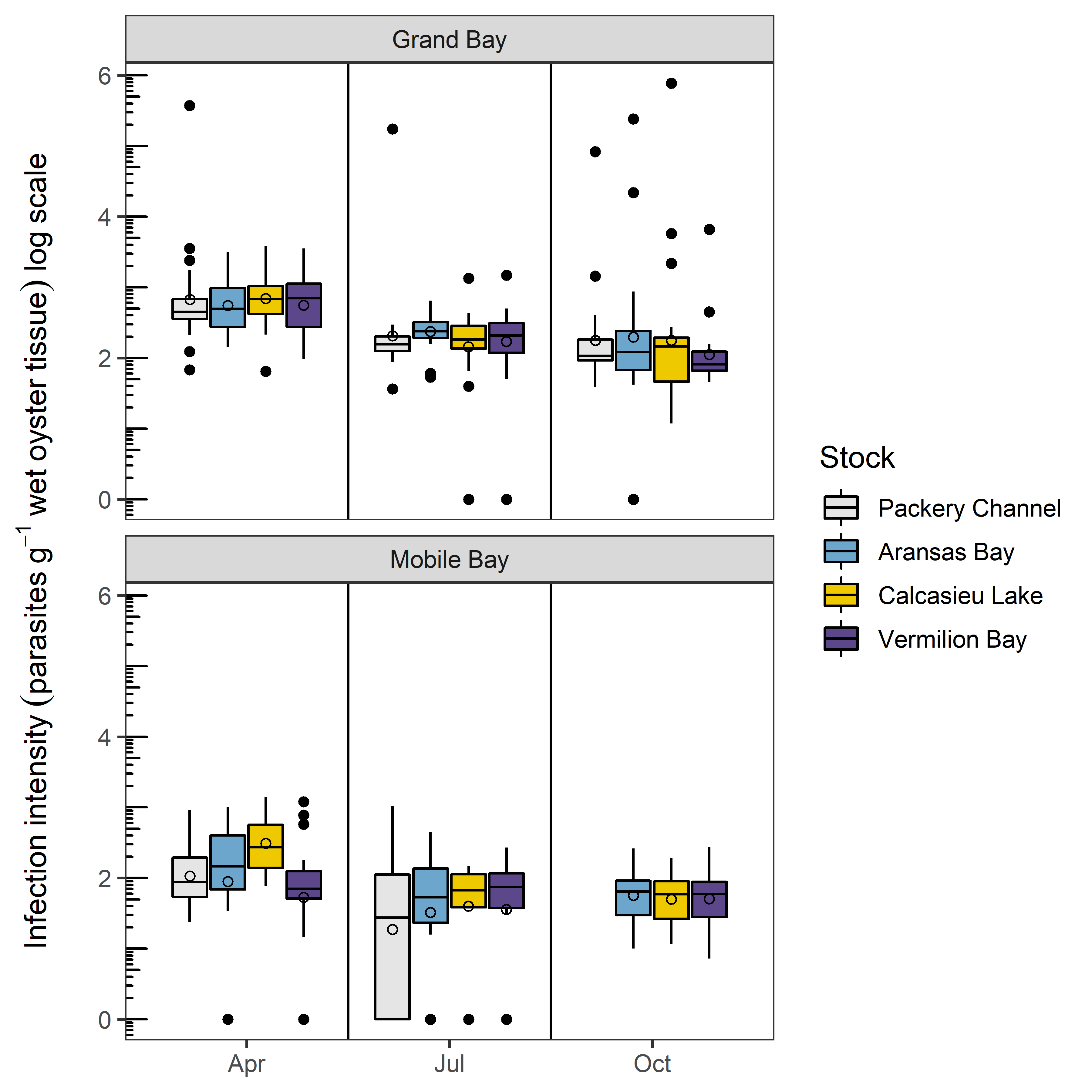


Supplemental Figure 7. *Perkinsus marinus* infection intensities of the progenies of the four oyster broodstocks at the Alabama Grand Bay and Mobile Bay sites sampled quarterly from April to October 2019. Box features are described in Supplemental Figure 6.
